# Brain homeostasis: VEGF receptor 1 and 2—two unequal brothers in mind

**DOI:** 10.1007/s00018-013-1279-3

**Published:** 2013-03-12

**Authors:** Ina M. Wittko-Schneider, Fabian T. Schneider, Karl H. Plate

**Affiliations:** 1 Neuroscience Center, Institute of Neurology (Edinger Institute), Goethe University Medical School, Heinrich-Hoffmann Strasse 7, 60528 Frankfurt, Germany; 2 Present Address: Institute for Stroke and Dementia Research, Klinikum der Universität München, Marchioninistrasse 15, 81377 Munich, Germany; 3 Present Address: Department of Biosciences and Nutrition, Karolinska Institutet, Novum, Hälsovägen 7, 14183 Huddinge, Sweden

**Keywords:** VEGF receptors, Neurogenesis, Neural stem cells, Brain development, Brain repair, Migration

## Abstract

Vascular endothelial growth factors (VEGFs), initially thought to act specifically on the vascular system, exert trophic effects on neural cells during development and adulthood. Therefore, the VEGF system serves as a promising therapeutic target for brain pathologies, but its simultaneous action on vascular cells paves the way for harmful side effects. To circumvent these deleterious effects, many studies have aimed to clarify whether VEGFs directly affect neural cells or if the effects are mediated secondarily via other cell types, like vascular cells. A great number of reports have shown the expression and function of VEGF receptors (VEGFRs), mainly VEGFR-1 and -2, in neural cells, where VEGFR-2 has been described as the major mediator of VEGF-A signals. This review aims to summarize and compare the divergent roles of VEGFR-1 and -2 during CNS development and homeostasis.

## Introduction

Cellular development is controlled in at least two different ways: by intracellular signal transduction pathways, and by external signals from cells in close vicinity. These extracellular signals are often growth factors secreted from neighboring cells, which comprise the “niche”, a specialized cellular microenvironment [1–3]. There is considerable evidence that, in cell types of the niche, an exceedingly wide diversity of cellular communication signals controlling developmental processes, like neurogenesis or gliogenesis, is mediated via receptor tyrosine kinases (RTKs) and their ligands [4–6]. Among those RTKs are the vascular endothelial growth factor receptors (VEGFRs), which were originally identified by their crucial role in development, maintenance and function of the vascular system [7–9]. These RTKs bind the members of the VEGF growth factor family, which contains six different homologues of dimeric glycoproteins, namely VEGF-A–E, and placenta growth factor (PlGF) [10, 11]. We and others have shown that VEGF-A, -B and -C exert versatile effects within the mammalian brain and stimulate neural cell proliferation, migration, differentiation and survival during development and in the adult [12–24]. In addition, neuronal cell survival-promoting properties of VEGF-A, -B, -C in central nervous system (CNS) pathologies, like stroke, Parkinson’s disease and amyotrophic lateral sclerosis (ALS), have been described in numerous studies in recent years (reviewed [25–28]). Thus, the VEGF/VEGFR-system is a promising therapeutic target for various CNS diseases and insults. Unfortunately, its role in both the vascular and the nervous system opens the door for detrimental side effects, like overriding endothelial cell growth or increased vascular leakage, leading to edema [29, 30].

**Table 1:**
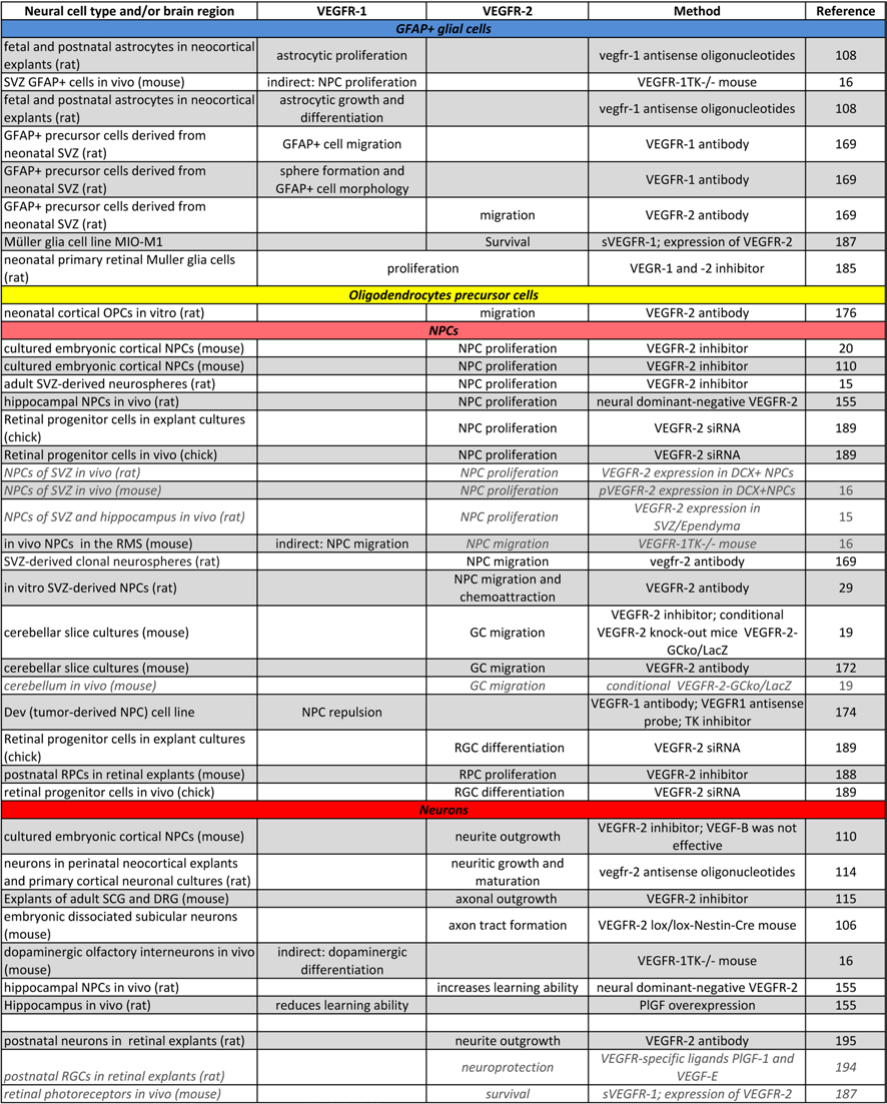
Comparison of the specific functions of VEGFR-1 and -2 in neural cells under physiological conditions

Shows the functions of VEGFRs in neural cells that have been proofed directly through loss-of-function experiments (normal black font) or strongly suggested (in italics, grey) by supporting data

Throughout life, vascular cells are close to CNS germinal zones and neural stem cells (NSCs), and neural precursor cells (NPCs) are always found within an angiogenic compartment [3, 31–33]. As the definition between NSCs and NPCs is not always identically used in the literature, we here use the term NS/PCs to cover both cell types. In the last decade, much effort has been expended to distinguish direct and indirect effects of the VEGF family members on CNS cells, especially on neurons, to circumvent these undesired outcomes of VEGF-based therapies. We and others have recently shown that VEGFR-1 and -2 are spatially and temporally expressed differently in the nervous system and carry out distinct roles within the CNS under physiological and pathological conditions (see sections below). These findings are of utmost importance as they provide the opportunity for the fine-tuning of therapeutic interventions in CNS protection and regeneration. Therefore, this review aims to illustrate the functional diversity of VEGFRs in the nervous system during brain development and maintenance under physiological conditions throughout life.

## The VEGFR-1 and -2 and their ligands

The main receptors for growth factors of the VEGF family are the RTKs: VEGFR-1 (fms-related tyrosine kinase-1; Flt-1) [7, 8], and VEGFR-2 (fetal liver kinase 1; Flk-1) [9] and VEGFR-3 (fms-related tyrosine kinase-4; Flt-4) [34, 35]. VEGFR-1, -2 and -3 are typical RTKs, which are composed of an extracellular immunoglobulin-like domain, a transmembrane domain, and an intracellular split TK domain. Ligand-binding to VEGFRs induces receptor homodimerization, followed by kinase activation and autophosphorylation of specific tyrosine residues, which in turn initiate multiple downstream signaling pathways [10, 11].

This review focuses on the divergent roles of VEGFR-1 and -2. The most well-studied ligand of the VEGF family, VEGF-A, acts predominantly through VEGFR-1 and -2 on neural and endothelial cells. Besides VEGF-A, VEGF-B and PlGF bind to VEGFR-1 and VEGFR-2 binds VEGF-C and VEGF-D [10, 11]. VEGFR-3, VEGF-C and –D will not be described in detail in this article, but in other articles of this issue. For a recent detailed review of VEGFR signaling cascades, see Koch and Claesson-Welsh [36].

### The VEGFR-1 and -2

VEGFR-1 expression, like VEGF-A, is inducible by hypoxia owing to a binding site for a hypoxia-inducible factor-1α (HIF-1α) in the VEGFR-1 promoter region. VEGFR-1 occurs in two isoforms: a membrane-bound form and a shortened soluble form (sVEGFR-1). The shortened form lacks the intracellular TK domain and is therefore incapable of intracellulary transduction of a VEGF-signal. This soluble form appears to act chiefly as a ligand-trap, thus having regulatory consequences for VEGF-mediated effects through other receptors. (reviewed [36, 37]). Overexpression of sVEGFR-1 reduces the binding of VEGF-A to VEGFR-2 and thereby diminishes VEGFR-2 signaling. This could be either indirectly by sequestering of the ligand or directly by formation of VEGFR-1/VEGFR-2 heterodimers [38–40].

In spite of its higher binding affinity to VEGF-A compared to VEGFR-2, VEGFR-1 shows poor autophosphorylation of the TK domain [41]. However, intracellular downstream target molecules of VEGFR-1 have been identified. Among them are phospholipase-Cγ (PlCγ), p38 mitogen-activated protein kinase (p38 MAPK), p85 phosphoinositide-3-kinase (p85 PI3K), and growth factor receptor-bound-2 (Grb-2) [37, 42, 43]. In vitro, VEGFR-1 seems to be directly involved in the control of endothelial cell migration, as the inhibition of VEGFR-1 in human umbilical vein endothelial cells (HUVECs) blocks their migration, but does not inhibit VEGF-A-induced proliferation. VEGFR-1 affects endothelial cell migration by modulation of the reorganization of actin filaments, favorably by p38 MAPK and Paxillin activation [44].

On vascular processes, VEGFR-1 seems to have an essential inhibitory role, since embryos that are homozygous for VEGFR-1-null mutation die early in embryonic development [embryonic day (E) 8.5] due to a disorganized vasculature with an elevated number of endothelial cells and overgrowth of blood vessels [45, 46]. This effect is subsequent to an increased mesenchymal–hemangioblast transition [47]. In line with this, transgenic mice in which the transmembrane domain and the intracellular domain of the VEGFR-1 are deleted display frequent embryonic death due to decreased development of the vasculature [48]. In contrast, the exclusive deletion of the intracellular signaling domain (VEGFR-1-TK^−/−^ mice) does not result either in embryonic lethality or in an obvious severe phenotype [49]. Nevertheless, VEGFR-1 signaling has been shown to play a role under certain pathological conditions, since pathological angiogenesis, e.g., tumor angiogenesis, is deteriorated when overexpressing the specific VEGFR-1 ligand PlGF-2 [50]. Moreover, in the brain of adult VEGFR-1-TK^−/−^ mice, we found an increased level of VEGF-A protein, which in turn enhanced olfactory neurogenesis and NPC migration [16]. Yet, the mechanism how VEGFR-1 signaling influences the amount of VEGF-A is still unclear.

Furthermore, VEGFR-1 seems to be important for the recruitment of VEGF to the membrane, as mice that express solely the soluble form of VEGFR-1 show high early embryonic lethality with a phenotype similar to the complete VEGFR-1^−/−^ knock-out mouse [48].

VEGFR-2 is the major mediator of VEGF-A-mediated trophic effects in the vascular system. VEGFR-2 signal transduction regulates the proliferation, migration and survival of endothelial cells and mediates signals increasing vascular permeability in response to VEGF-A (reviewed [27, 28, 51]). Furthermore, VEGFR-2 promotes VEGF-C-induced lymphangiogenesis. VEGFR-2 knock-out mice fail to develop blood islands, and do not form a proper vascular system, leading to early embryonic death between E8.5 and 9.5 [52, 53]. Unlike VEGFR-1 and VEGF-A, VEGFR-2 expression is not regulated by hypoxia, but is upregulated by VEGF-A [54].

Similar to the vascular system, in the CNS most of the trophic effects of VEGF-A have also been ascribed to VEGFR-2 signaling. VEGFR-2 has a high kinase activity and activates several downstream pathways, like the PI3-Kinase/-pathway or PLC-γ-pathway. Those pathways trigger proliferation and diminish apoptosis of endothelial and neural cells. VEGFR-2 signaling further promotes proliferation, migration and neuronal differentiation of NPCs (see sections below for details).

Similar to sVEGFR-1, a truncated soluble form of VEGFR-2 (sVEGFR-2) occurs physiologically and functions as an endogenous antagonist of VEGF-C, negatively regulating, e.g., lymphangiogenesis [51]. Astonishingly, native sVEGFR-2 has a poor affinity to VEGF-A and lacks anti-hemangiogenic effects antagonizing VEGF-A [51].

VEGFR-1 and -2 show additional binding affinities to non-TK receptors Neuropilin (Nrp)-1 and Nrp-2, which are likewise receptors for both VEGFs and Semaphorins. Nrps are involved in the control of the development of the neural, the lymphatic and the vascular systems and especially of axonal guidance (reviewed [55]). Nrps act merely as co-receptors and seem not to possess intrinsic catalytic activity [56]. Nrp-1 and -2 form complexes with VEGFR-2 and thereby fortify VEGF-A-induced VEGFR-2 activity [57–59]. Nrp-1 also binds with high affinity to VEGFR-1, which counteracts an interaction of VEGF-A and Nrp-1 and finally results in reduced VEGF-A effectiveness [59]. This is yet another level of control of VEGF-A dosage. Besides VEGF-A isoforms, Nrp-1 binds to VEGF-B, VEGF-E, and PlGF, while Nrp-2 additionally has binding affinity for VEGF-C and PlGF-2 (reviewed [10, 11, 36]). Phenotypic analysis of overexpression and knock-out mouse models of Nrps revealed that they are important regulators of the development of the vascular and the lymphatic systems as well as of the CNS [60–62].

### The VEGF family growth factors

VEGF-A has firstly been described as a vascular permeability factor (VPF) in tumors and has been considered for a long time as an endothelial cell-specific mitogen [63]. Six Isoforms of VEGF-A are generated by alternative splicing, ranging in length from 121 to 206 amino acid residues (VEGF121–206; reviewed [64]). The three major isoforms are VEGF121, 165, and 189 in humans and VEGF120, 164, and 188 in mice. These isoforms differ in their binding affinity to extracellular matrix proteins and therefore show distinctions in their diffusion ability [65]. VEGF-A is a mitogen for endothelial cells and a survival-promoting factor for newly formed vessels that is indispensible for the development of the vascular system [25]. In mice, even the deletion of only one allele of the *Vegf*-*a* gene leads to embryonic lethality on day E9, due to a misbuilt vascular system of the whole embryo and the yolk sac [66–68]. In the embryo, gradients of VEGF-A function as guidance cues for forming vessels [65, 69, 70]. Likewise, these gradients are critical for brain development, as shown by Raab et al. [71] in a mouse model of specific VEGF deletion in neuroectodermal cells. Recently, Lee et al. [72] showed an endothelial expression of VEGF-A in the adult brain and revealed an autocrine effect on vascular homeostasis.

Expression of VEGF-A protein is regulated by many factors, among them hormones, growth factors, and oxygen concentration. Hypoxia-inducible factors (HIF1α and HIF2α) bind to a hypoxia response element (HRE) in the 5′ *Vegf*-*a* promoter-region, thereby inducing VEGF-A expression [73–75]. Hence, VEGF is upregulated and additionally promotes its trophic effects on endothelial and CNS cells following hypoxic conditions, e.g., during tissue growth in development or tumors [76, 77]. VEGF-A synthesis is strongly upregulated after various pathological insults [78, 79]. Genetic mutation of the HRE of the *Vegf*-*a* gene (VEGFδ/δ mice) decreases VEGF-A protein concentrations and leads to an adult-onset motor neurodegenerative phenotype, resembling ALS [80]. Thus, VEGF-A is of great significance for therapeutic interventions in various pathological settings.

VEGF-B occurs in two isoforms and shows a great homology to VEGF-A [81, 82]. Like VEGF-A, VEGF-B stimulates proliferation of endothelial cells in vitro and angiogenesis in vivo, although to a much lower extent than VEGF-A and confined to certain conditions [81, 83]. In contrast to VEGF-A, VEGF-B is dispensable for embryonic vascular development. Mice bearing a homozygous deletion of the *Vegf*-*b* gene (VEGF-B^−/−^) are viable and fertile. Nonetheless, these animals exhibit disturbances in cardiac development and function [84, 85]. Moreover, animal models have shown a role for VEGF-B in vascular remodeling under pathological conditions [86].

The promoter region of the *Vegf*-*b* gene lacks an HRE and therefore, unlike VEGF-A, the expression of VEGF-B is not regulated by hypoxia [87]. Though VEGF-B is not essential for angiogenesis in most tissues and settings, it has been demonstrated to be a crucial survival factor for blood vessels [88, 89]. Due to the fact that VEGF-B shows only minor angiogenic activity, VEGF-B was shown to have a better safety profile as a neuroprotective survival molecule than VEGF-A and might therefore be a good therapeutic target for neurodegenerative diseases [90].

PlGF has been discovered by its angiogenic role in the placental chorion and the maintenance and development of the human placenta [91]. Three isoforms of PlGF exist in humans, but only one isoform (PlGF-2) has been found in mice [92]. Carmeliet et al. [93] showed that, whereas a genetic deletion of *Plgf* (PlGF^−/−^) in mice did not alter embryonic angiogenesis, it diminished vessel growth in pathological situations. In compliance with this, overexpression of *Plgf* increased angiogenesis and vessel permeability [94]. Furthermore, genetic ablation of PlGF delays angiogenic reaction to hypoxia, and the accumulation of fibrinogen in microvessels of the brain [95], and thereby indirectly affects neuronal survival.

The phenotypes resulting from genetic deletion of all VEGF family members and VEGFRs in mice have been nicely summarized and depicted in a review by Olsson et al. [37].

## The neurobiology of VEGFR-1 and -2

**Fig. 1:**
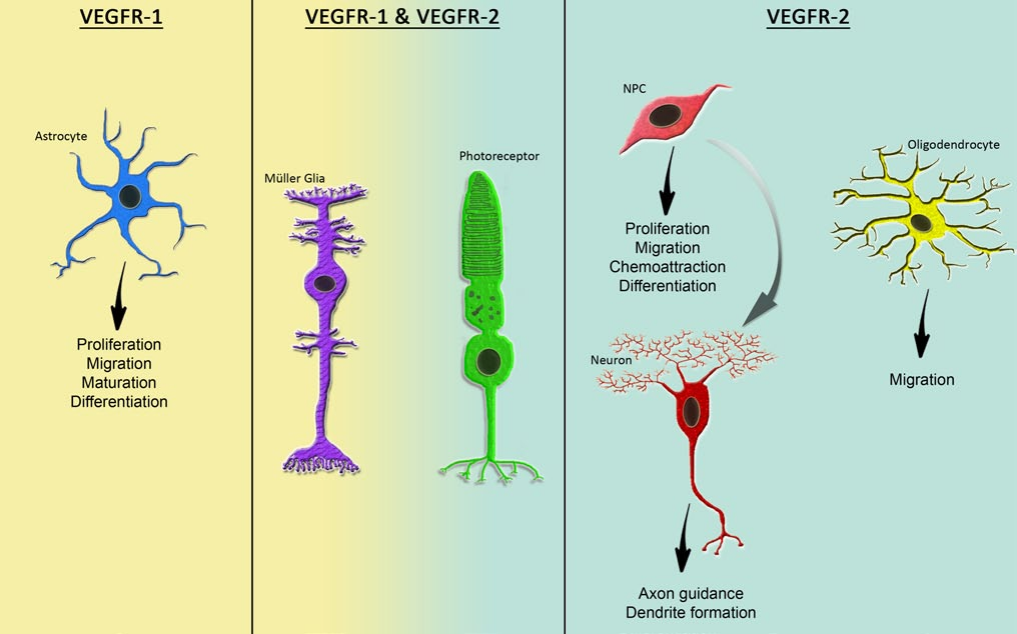
VEGFR-1 and -2 are expressed differently and exert diverse functions in distinct cell types of the nervous system under physiological conditions. Whereas VEGFR-2 appears to be the main receptor transmitting mitogenic effects in NPCs and differentiated neuronal cells, VEGFR-1 seems to be the main receptor facilitating glial development and survival. Both VEGFRs mediate VEGF-induced neuroprotection and Müller glia cell survival in the adult retina

Although the role of VEGF family members in neural cells has been studied extensively within the last decade and VEGF-A has been demonstrated to be involved in many steps of nervous system development and function, the downstream signaling pathways and the roles of VEGFRs are yet not fully understood. VEGFs have been shown to affect NS/PCs differently in specific areas of the brain and at various stages of development. Great efforts have been made to explore and discriminate the direct and indirect neurotrophic effects of VEGFs and to define the mediating receptors in neural cells. Here, we portray and compare the physiological functions of VEGFR-1 and -2 in the developing and the adult nervous system. Table 1 summarizes the diverse functions of both VEGFRs in neural cells. Their functions are further illustrated in Fig. 1.

### VEGFR-1 and -2 are regulators of brain development

The development of the mammalian brain is based on the presence of undifferentiated, multi-potent NSCs that give rise to all neural cell types: (1) NPCs that can differentiate into all neuronal cell types of the brain and (2) glial precursor cells, which generate astrocytes and oligodendrocytes. In the embryo, the first NSC populations are located in the ventricular zone (VZ), the multi-layered wall of the primordial ventricles that later gives rise to the subventricular zone (SVZ) and bears neurogenic potential throughout life. With the start of neurogenesis, multi-potent, self-renewing radial glia develop from neuroepithelial cells situated in the wall of the neural tube. Radial glia cells are present in great numbers at the peak of embryonic neurogenesis and serve as NSCs of the embryonic brain, but also function as migration scaffold for cortical precursors. In the course of the development, the NSC number declines. Later, a defined subgroup of radial glia transforms into a subpopulation of astrocytes, which serve as NSCs in postnatal and adult age (for detailed review, see [96–99]).

#### Expression of VEGFs and VEGFRs 1 and 2 in the developing brain

##### VEGF family ligands in the developing NS

 In the embryonic brain, neuroectodermal cells of the VZ express high levels of VEGF-A [100]. The resulting VEGF-A-gradient from the VZ to the brain parenchyma most likely directs invading vessels/endothelial cells expressing VEGFR-1 and -2 [100, 101]. Homozygous deletion of *Vegf*-*a* in Nestin-expressing (Nestin+) cells, i.e. primarily NS/PCs surrounding the lateral ventricles, results in disruption of angiogenesis and thereby reduces vascularization of the embryonic mouse brain [71, 102]. In addition, the loss of secreted VEGF-A leads to extensive apoptosis of the cells surrounding the lateral ventricles. These transgenic mice display a phenotype that resembles human microencephaly, i.e. a severely reduced growth of the forebrain and a strong flattening of the skull [71, 102].

After birth, neuronal expression of VEGF under physiological conditions has been described but is controversial. Ogunshola et al. [32] showed neuronal VEGF-A in early postnatal stages. Later, postnatally, between P13 and P24, when angiogenic activity in the brain is highest, the expression of VEGF-A changes to a primarily glial expression pattern. Glial expression of VEGF-A has previously been linked to postnatal neurogenesis in the cerebellum [103]. Furthermore, VEGF-A is expressed in Purkinje cells during postnatal development and is found in a radial concentration gradient from the Purkinje cell layer to the upper layers, corresponding to its chemoattractive function [19]. This points to an intertwining of brain angiogenesis and VEGF expression in neurons and neuronal metabolism [32].

VEGF-B mRNA is highly expressed in the murine embryonic CNS and spinal cord. Lagercrantz et al. [104] reported even higher transcript levels of VEGF-B than VEGF-A in the developing mouse brain.

##### VEGFR-1 and -2 in the developing NS

 Inconsistent results of VEGFR-1 and -2 expression in the developing CNS have been published. During embryonic development, the mRNA of VEGFR-1 and -2 VEGFR-2 has been typically shown in brain endothelial cells of the murine brain, but it has also been detected in the embryonic forebrain (E15) on neural cells, which simultaneously express VEGF-A. This is indicative of both paracrine and autocrine effects of VEGF-A in the developing brain [105]. In addition, expression of VEGFR-2 and Nrp-1 has been demonstrated in subiculo-mamillary axons of the developing hippocampus [106]. Furthermore, in vitro, VEGFR-1 and -2 expression has been shown in NS/NPCs [20]. Both VEGFR-1 and -2 have been detected in cultured Nestin+ SVZ-derived NS/PCs from newborn rats, which do not express markers of differentiated neurons, glial or endothelial cells [107]. In cortical explants of neonatal rats, VEGFR-1 is expressed by astrocytes, where it mediates VEGF-induced gliotrophic effects [108].

In the postnatal brain, a strongly regionalized expression of VEGR-1 in neurons of the cortex, striatum, and hippocampus within the first weeks after birth has been described [109]. This neuronal expression decreases with age. At P30, only a few neurons reveal VEGFR-1 immunoreactivity, whereas at P90, VEGFR-1 expression is no longer detectable in neurons [109]. A similar switch of cell-type-specific expression in early postnatal development has also been described for VEGF-A (see above). In contrast to VEGFR-1, according to Yang et al. [109], the number of mature neurons, which express VEGFR-2, grows with age and expression is evenly distributed without regional differences.

#### Divergent roles of VEGFR-1 and -2 in developmental neurogenesis

Until now, the role of VEGFRs in embryonic CNS cells has not been entirely clarified. Whereas a reduction of VEGF-A results in strong defects in brain development mainly due to impaired vascularization, neither the genetic deletion of VEGFR-2 in Nestin+ cells and the deletion of VEGFR-1-signaling in mice show any severe developmental phenotype [49, 71, 102]. Thus, VEGFR-1 and -2 signaling in neural cells does not seem to be essential for CNS development. Further studies dissecting indirect and direct effects of VEGFs on neural cells and the involvement of VEGFRs in vivo are difficult due to the early embryonic lethality of the knock-out mice [46, 53] and the concomitant changes in vascularization when modulating the VEGF/VEGFR system.

##### In vitro

 Most studies unraveling VEGFR signaling in cells of the developmental NS have used in vitro systems. Although VEGFR-2 is regarded as the main mediator of VEGF-A-induced direct neurotrophic effects, effects of VEGF-B, which only acts through VEGFR-1 on NS/PCs, have also been reported. In embryonic cortical cultures, VEGF-A and VEGF-B dose-dependently increased proliferation, whereas PlGF had no effect [21, 110, 111]. Jin et al. [110] reported that VEGFR-2, but not VEGFR-1, is expressed in these NS/PCs and that inhibitors of VEGFR-2 blocked the VEGF-A-induced effect. From the same laboratory, Sun et al. [21] showed that in this in vitro model VEGF-B treatment increased newborn cells that express markers of early NS/PCs (here Nestin), but not of differentiated neurons. Unfortunately, no information about the VEGFR expression was given in the latter publication. As VEGF-B only binds to VEGFR-1, but not -2, it is unclear how this effect is mediated. Both ligands, VEGFA and VEGFB, have binding affinities to Nrp co-receptors, but Nrps seem not capable of autonomous signal transduction and need to form complexes with other RTKs [36, 56, 112].

In primary cultures of cortical neurons derived from newborn rats (P1) VEGFR-1 and -2 were expressed and, surprisingly, the expression of VEGFR-2, but not -1, was amplified in response to the selective VEGFR-1-ligand. PlGF acted neuroprotectively in these cultures in an in vitro model of cerebral ischemia, and this might secondarily be mediated by VEGFR-2 [113]. Future studies using inhibitors of VEGFR-1 and -2 or genetic ablation of these RTKs are needed to unravel the intertwined neurotrophic signaling cascades of these receptors.

Besides the transduction of mitogenic signals of VEGF-A, VEGFR-2 mediated VEGF-A-induced neuritic growth and maturation in an organotypic perinatal rat cortical explant model. In this process, the PI3-K/Akt and the MAPK/extracellular signal-regulated kinase (ERK) pathways were activated downstream of VEGFR-2. In primary neuronal cultures, VEGF-A increased neuronal cell body size and stimulated neurite formation via VEGFR-2 signaling [114]. In fetal and postnatal rat cortical organotypic explant cultures, VEGFR-1 has been shown to transduce VEGF-A-stimulated astrocytic proliferation. Here, the MAPK/ERK and PI-3 kinase signaling pathways were also identified as downstream signaling pathways. Ectopic VEGF enhanced expression of bFGF (basic fibroblast growth factor), which is a stimulating factor for NPCs [108]. This shows once more the potential interplay of the differentially expressed VEGFR-1 and -2 in the regulation of neurogenesis.

Also, in the developing PNS, VEGF-A shows neurotrophic properties. In cultured murine superior cervical ganglia and DRG, VEGF-A increased survival and neuritic outgrowth. The latter effect has been shown to be mediated by VEGFR-2 [115].

##### In vivo

VEGFR-2 has been demonstrated to employ a function in the development of the hippocampal axon tract, which plays an important role in memory and emotional behavior. Genetic deletion of VEGFR-2 in Nestin+ cells altered embryonic development of the postcommissural fornix pathway and induced fiber loss of the postcommissural axons that project from the hippocampus to the hypothalamus. These changes, albeit less prominent, was not entirely compensated for during development and persist at least into juvenile age (P30) [106]. In vitro, the VEGFR-2 signaling effect on axon growth occurred in response to Sema3A, but independent of VEGF-A, by forming a receptor complex with Nrp-1 and PlexinD1, which activated the PI3 K/Akt pathway [106]. A similar effect has been observed in *Drosophila*, where genetic ablation of the PDGFR/VEGFR homolog resulted in developmental defects of the commissural axons. These effects are preceded by survival and migration deficits in midline glial cells. Here, the effect on midline glia was transmitted via the Akt and ERK pathways [116].

In summary, VEGFR-1 and -2 transmit distinctive, non-overlapping signals of VEGF family members in prenatal/perinatal neural cells. VEGFR-2 seems to be the major receptor transmitting mitogenic and trophic signals in NS/PCs and neurons as well as neurite growth and axon pathfinding cells, whereas VEGFR-1 signaling mediates glial cell proliferation and survival. Additional cell-type-specific and inducible knock-out animal models are needed to investigate the roles of VEGFRs in nervous system development disconnected from the vasculature.

### VEGFR-1 and -2 exert distinct roles in adult neurogenesis

**Fig. 2:**
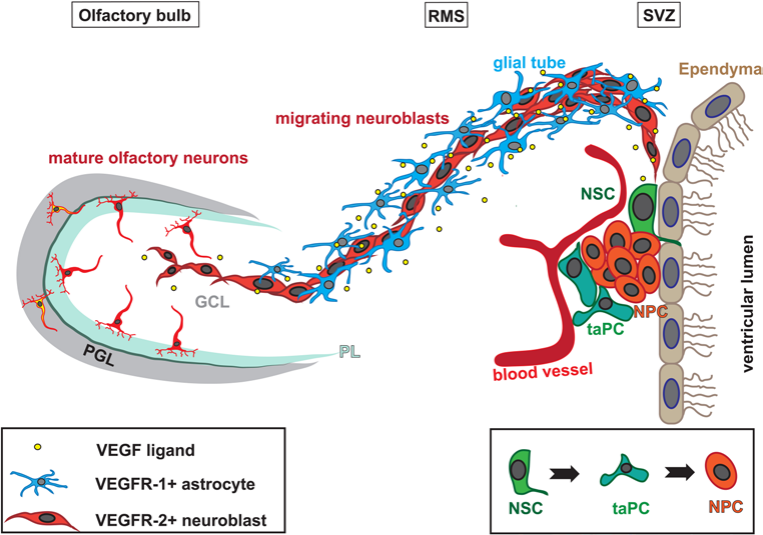
Schema of olfactory neurogenesis. Subpopulations of GFAP+ cells serve as neural stem cells in the SVZ (*NSC*, *blue*). These generate transit-amplifying neural progenitor cells (taPC; *green*), which in turn produce neuronal progenitor cells (*NPC*; *red*). NPCs migrate in a chain migration mode along the* RMS* towards the* Olfactory bulb*. The RMS is ensheathed by glial cells (*dark blue*), which secrete regulatory factors, like VEGF-A and BDNF (*yellow*) that influence the migration of the NPCs. Once arrived at the olfactory bulb, the NPCs detach from the chains and migrate individually, radially in the granular cell layer (*GCL*). A minority of cells migrate further outwards through the plexiform layer (*PL*) to the periglomerular cell layer (*PGL*)

In the adult mammalian brain, physiological/spontaneous neurogenesis is mainly confined to two regions: the SVZ of the lateral ventricles and the dentate gyrus of the hippocampus [117–120]. In the SVZ NSCs, which are GFAP-positive slow cycling cells, are located mainly in the lateral wall of the lateral ventricles [121, 122]. Most of the new olfactory neurons in the adult murine brain originate from there [120, 123]. GFAP+ NSCs in the adult SVZ generate active, rapid transit amplifying cells (taPCs) typically found in clusters [122], which in turn produce NPCs that migrate through the RMS to the OB [120, 123, 124]. Unlike the radially migrating neurons in the developing cerebral cortex [125], the adult SVZ-derived NPCs retain their proliferative activity and migrate in a chain migration mode [126] (Fig. 2).

NPC migration is tightly regulated. Glial cells secrete regulatory factors for neuronal guidance [127–129]. In the RMS, migrating NPCs are enwrapped by a glial tunnel constructed of specialized astrocytes and extracellular matrix proteins [130, 131]. On arrival in the olfactory bulb (OB), the NPCs detach from their chains and migrate as single cells radially towards their final position, where they differentiate into distinctive neuronal subtypes and functionally integrate into the existing network [132, 133] (Fig. 2). Most cells remain in the granular cell layer and adopt a GABAergic phenotype, but few (5 %) migrate towards the outer layer, the periglomerular layer, where they differentiate into GABAergic or dopaminergic interneurons [132–134]. Until now, it has not been entirely clear if the NPCs are already primed to develop into a specific interneuron subtype in the SVZ or if determining signals define their latter phenotype during their migration through the RMS or even at their final location within the OB [135].

Recent results suggest that NS/PCs itself may possess not only the potential to replace lost cells but may additionally have a beneficial effect on the surrounding tissue [136, 137]. Consequently, the mechanisms that control NS/PC behavior and the NSC state are of high therapeutic relevance.

#### Different expression patterns of VEGFR-1 and -2 in the adult rodent NS

##### VEGF family ligands in the adult rodent CNS

**Fig. 3:**
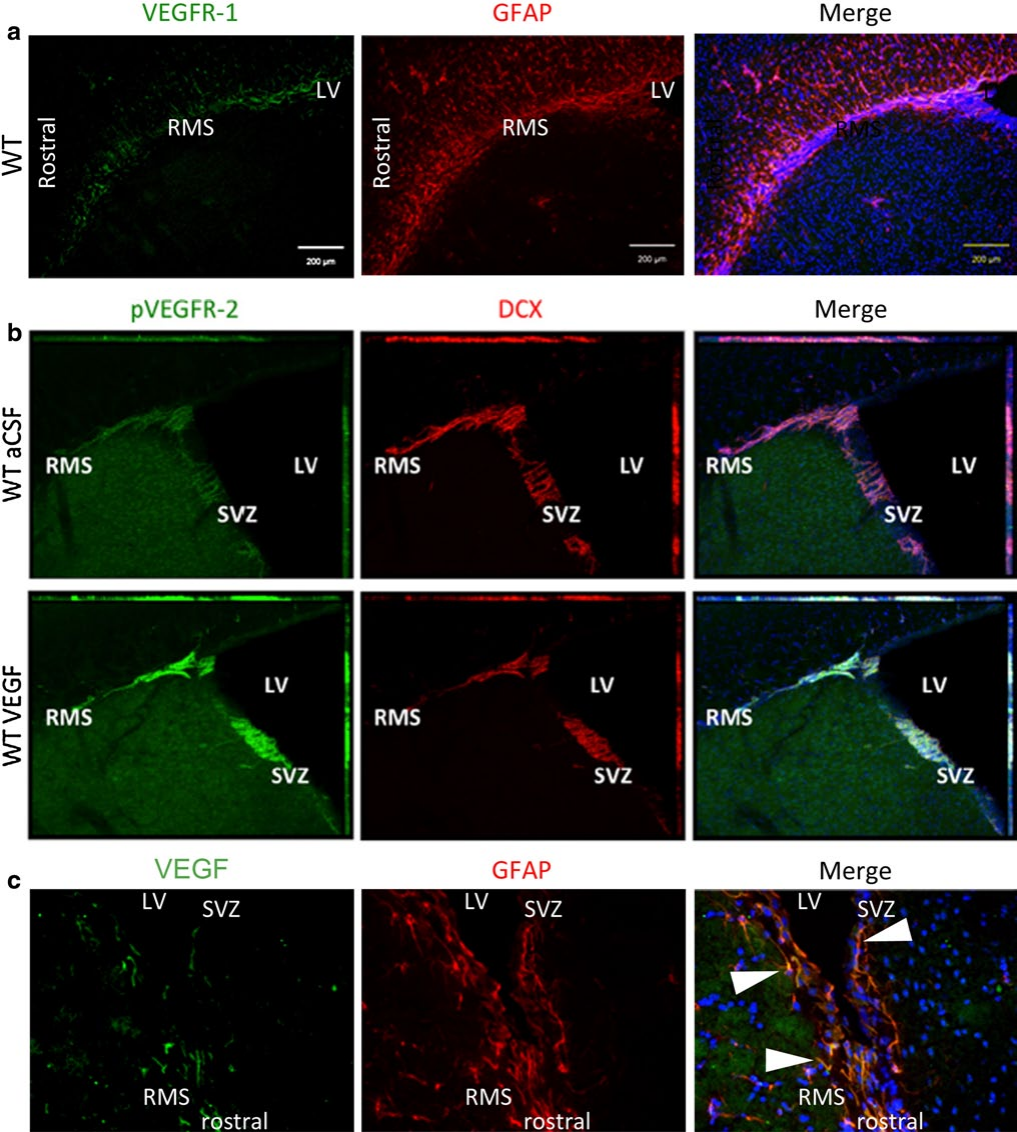
Expression of VEGFR-1 and -2 and ligand VEGF-A in the SVZ-RMS region of the adult brain. Immunostained sections from adult mouse brains show that, whereas VEGFR-1 is expressed in GFAP+ cells of the SVZ and RMS (**a** sagittal section) we detected pVEGFR-2 in DCX+ NPCs in these regions. The expression of pVEGFR-2 was increased after intracerebroventricular injection of VEGF-A protein (**b** coronal section; adapted with permission from *Journal of Neuroscience* from Wittko et al. [16]; doi:10.1523/JNEUROSCI.5527-08.2009). The ligand VEGF-A was found on SVZ and RMS glial cells and within the choroid plexus (**c** sagittal section). *White arrows* point to GFAP/VEGF-A double-labeled cells

 In the adult rodent, a low level of VEGF expression has been observed throughout the entire brain, with the highest expression levels in the regions of canonical adult neurogenesis, in the plexus choroideus, and in the cerebellum [15, 19, 100, 138–142]. From the choroid plexus, VEGF-A is secreted into the liquor and can consequently act directly on ependymal and subventricular cells that express VEGFRs. Balenci [139] reported VEGF-A expression in cells expressing the glial marker GFAP (glial fibrillary acidic protein; Fig. 3). Interestingly, glial cells of the SVZ and the RMS showed a specific elevated expression in comparison to glial cells of other regions [139]. VEGF-A mRNA and VEGF-A protein were furthermore detected in neurons, i.e. mitral cells and juxtaglomerular cells (periglomerular and tufted cells) as well as in astrocytes of the olfactory bulb (OB) [17]. Moreover, in the hippocampus, VEGF-A is expressed in granule neurons and in proliferating cells that reside close to blood vessels, pointing to an direct mitogenic effect of VEGF-A on NS/PCs [14]. Several studies have shown that VEGF-A is expressed by cultured neural stem cells/neural progenitor cells (NS/PCs) from hippocampus, SVZ, and OB [15, 141, 142].

In the adult cerebellum, VEGF is expressed in Purkinje cells, Bergmann glia, and in astrocytes in the internal granular layer [19].

VEGF-B is expressed in all vessels and the choroid plexus as well as in neurons of the adult rodent brain [143–145], and its expression is upregulated following brain injury [144]. In rat midbrain cultures, VEGF-B is expressed in all neurons, including dopaminergic neurons, and acts neuroprotectively in an in vitro model of Parkinson’s disease [146]. VEGF-B is further expressed by mouse motor neurons and dorsal root ganglion (DRG) neurons, and promotes motor neuron survival in an autocrine mode. Astrocytes may upregulate VEGF-B after injury to retain neuronal survival in a paracrine manner [147]. In addition, VEGF-B mRNA was found to be highly expressed in the retina, where it also exerts a survival-promoting role [144]. Due to the fact that VEGF-B shows only minor angiogenic activity, VEGF-B has been shown to have better safety profile as a neuroprotective survival molecule than VEGF-A, [90] and might therefore be a good therapeutic target for neurodegenerative diseases.

Whereas in adult mice PlGF is expressed in neurons, vessels and astrocytes throughout the brain, and is upregulated after ischemic injury, it has not been detected in rat brains under physiological conditions [148]. In the peripheral nervous system (PNS), in sciatic nerves, and lumbar DRGs, PlGF is expressed in neuronal cells, but neither in myelating Schwann cells nor in endothelial cells [149].

##### VEGFR-1 and -2 in the adult rodent CNS

 The VEGFR-1 and -2 are expressed in different patterns in regions of spontaneous adult neurogenesis. The endothelial expression of VEGFR-1 and -2 seems to be related to the angiogenic activity in the tissue. Under physiological conditions, there is practically no angiogenic activity in adult brain, and VEGFR expression in brain endothelial cells is strongly reduced [109], but can be (re-)activated in response to injury [79, 148]. Nevertheless, a recent study reported VEGFR-2 and VEGFR-1 mRNA in endothelial cells of the adult hippocampus [18].

Many GFAP+ cells in the SVZ and the OB strongly express VEGFR-1, as well as fewer cells in the hippocampus (Fig. 3) [16]. In the adult SVZ, GFAP + cells have been reported as NSCs, which generate mainly neuroblasts [122]. However, the VEGFR-1+/GFAP+ cells detected in the adult mouse brain were not proliferative themselves but often located in ultimate proximity of vessels and proliferative DCX+ NPCs[16]. This suggests that VEGFR-1 is part of the glia-vascular neurogenic niche, which influences NS/PC development. Endothelial cells and glial cells, especially astrocytes, secrete trophic factors, like VEGF-A, FGF-2, bbrain-derived neurotrophic factor (BDNF) and others, that support the maintenance of the stem cell state, promote self-renewal of NCS and/or trigger neuronal determination, migration and differentiation (reviewed [127]). Adult SVZ-derived NPCs migrate through the RMS through a glial tunnel, which is built of specialized astrocytes and extracellular matrix components [130, 150]. VEGFR-1 is expressed in the GFAP+ cells along the entire RMS and in the bordering corpus callosum of the adult mouse brain (Fig. 3) [16]. VEGFR-1 is also expressed in activated astrocytes exposed to infused VEGF-A in vivo [151]. In contrast, VEGFR-2 is induced in neuronal and endothelial cells by infused VEGF-A or injury [151]. Furthermore, both VEGFR-1 protein and mRNA have been shown in neurons in the pyramidal cell and the granule cell layer of adult rat hippocampus [152].

Expression of VEGFR-2 has been shown in ependymal cells of the LVs [15, 109] and in DCX+ NPCs of the hippocampal subgranular zone and the SVZ of adult rats [20]. Phosphorylated (p)VEGFR-2 was detected in DCX+ NPCs of the SVZ and RMS and is elevated after intracerebroventricular infusion of VEGF-A (Fig. 3). Levels of pVEGFR-2 are similarly boosted in VEGFR-1TK^−/−^ mice, which exhibit higher concentrations of VEGF-A protein in their brain tissue. Intriguingly, pVEGFR-2 has not been found in the OB in these conditions/animals [16]. Thus, VEGFR-2+ NPCs seem to lose their phosphorylated state when entering the OB [16].

Furthermore, migrating cerebellar granular cells express VEGFR-2, which mediates VEGF-A-induced chemoattraction [19].

##### VEGFR-1 and -2 in adult rodent CNS cells in vitro

 The regional and cellular heterogeneity of VEGF receptor expression in areas of spontaneous adult neurogenesis implies distinct functions of both receptors in the regulation of adult neurogenesis and CNS maintenance. VEGFRs may contribute to the region specific properties and cell fate of adult CNS stem cells. This is supported by the diverse expression pattern of VEGF receptors that has been demonstrated in cultured adult NSCs derived from different origins (SVZ, OB, hippocampus) and which changes in response to VEGF-A [15, 142]. In vitro data of VEGFR expression differ greatly between studies analyzing different brain regions, time-points of development or animals and also between laboratories. Expression of VEGFR-2 and VEGF-A protein, but not vegfr-1 mRNA, have been shown in NS/PCs isolated from juvenile rat SVZ [142], whereas mRNAs of VEGF-A, VEGFR-1 and VEGFR2 mRNA were detected in adult mice SVZ-derived NS/PCs [141]. Supplementary, VEGFR-1 and -2 protein were revealed in cultured NS/PCs generated from the SVZ of newborn rats [107].

##### VEGFR-1 and -2 in the adult rodent PNS

 VEGFRs 1 and 2 are not only expressed in neural cells of the brain. Poesen et al. [147] demonstrated VEGFR-1 expression also in spinal motor neurons and astrocytes of wildtype and pre-symptomatic adult mice overexpressing a mutated human superoxide dismutase-1 gene, which serves as a mouse model for ALS. Furthermore, primary embryonic motor neurons and astrocytes, express VEGFR-1 and VEGF-B, implying an autocrine neurotrophic effect [147]. Likewise, VEGFR-1 and -2 are constitutively expressed in neurons, vascular endothelial cells and some astrocytes of the spinal cord [153]. Besides this, VEGFR-2 expression has been found in cultured superior cervical ganglia and DRGs of adult mice [13].

##### VEGFR-1 and -2 in the adult primate brain

 In contrast to the expression pattern in the adult rodent brain, VEGFR-1 is expressed in proliferating cells and in Nestin+ cells of the SVZ of the adult primate brain. In this context, VEGFR-2 is mainly detectable in the vessel walls, predominantly in endothelial cells. Astonishingly, VEGFR-1 has not been detected in the primate RMS [154]. Possibly the glial tunnel and so also VEGFR-1 signaling is not of comparable importance in the primate brain, due to the brain anatomy and to the facts that migration distances are relatively much shorter than in the rodent and that olfactory neurogenesis is of much lower significance in primates and humans.

#### Generation of new neural cells from adult NPCs in vitro

Cultured NS/PCs isolated from the regions of adult spontaneous neurogenesis express VEGF-A and stimulate adult NS/PC proliferation dose-dependently in an autocrine manner via VEGFR-2. Effective doses vary widely between studies. Schänzer et al. demonstrated a maximal stimulation at 50 ng/ml VEGF-A [15]. In contrast, Meng et al. [141] reported that low doses of VEGF-A (here, 50 ng/ml) upregulated the expression of VEGFR-1 and -2 in adult SVZ-derived neurosphere cultures, but did not alter cell proliferation or differentiation, whereas very high amounts of VEGF-A (500 ng/ml) induced a decline of VEGFR expression accompanied by an amplified proliferation of NS/PCs and neuronal differentiation. Furthermore, high doses of VEGF-A expanded GFAP+ cells [141]. Unfortunately, it has not been shown if VEGFR-1 is directly involved in GFAP+ cell proliferation or survival in these experiments. The fact that high doses of VEGFA reduce VEGFR expression, but simultaneously induce neurotrophic effects, let Meng et al. [141] hypothesize that another receptor, i.e. Nrp that interacts with VEGFRs and modulates VEGF-A signals in NS/PCs, may be responsible for the signal transduction in these cells. An autocrine effect of VEGF-A on NS/PCs in vitro has been demonstrated by blocking of VEGFR-2 with an specific inhibitor [15].

So far, no direct influence of PlGF on the adult CNS under physiological conditions has been reported. However, PlGF has been shown to act neuroprotectively on astrocytes and neurons in vitro [95].

#### Generation of new neurons in the adult NS in vivo

Although considerable efforts have been spent analyzing downstream pathways and identifying direct and indirect neurotrophic effects of VEGFs on adult neural cells, it has been very difficult to define the mediating receptor in vivo and to exclude secondary effects, due to the vast number of cell types that respond to VEGF family members.

##### VEGFR-1 and -2 control proliferation, differentiation and survival of SVZ-derived NS/PCs

 In vivo, the intracerebroventricular infusion of VEGF-A with osmotic mini-pumps into adult rats at a low dose (2.4 ng/day) increased the quantity of neurospheres generated from the lateral ventricle wall and the number of new neurons in the OB and the hippocampus. This boost in neurogenesis neither resulted from an enhanced proliferative activity of NS/PCs in esponse to VEGF-A nor was it accompanied by changes in the vasculature, but was due to improved survival of NS/PCs [15]. In contrast, another study reported a proliferation-promoting effect of ectopic VEGF-A, but no decrease in apoptosis [20]. In the latter study, a higher dose of VEGF has been used and vascular effects have been observed. Similar to the in vitro situation, effects of VEGF-A on adult neurogenesis seem to be dose-dependent in vivo. Unlike in cultured cells, in vivo the involvement of VEGFR-2 in VEGF-A induced adult neurogenesis has not yet been clearly shown.

Recently, we could show for the first time that endogenous VEGF-A stimulates neurogenesis in the adult mouse brain. Proliferation of DCX+ NPCs was amplified in the SVZ, but not in the RMS of VEGFR-1-signaling-deficient mice that exhibit elevated VEGF-A protein levels [16]. Contrastingly, the number of non-neuronal cells in neurogenic regions was not increased by infused VEGF-A in the rat [15]. This was supported by our study, which showed that elevated levels of VEGF-A in VEGFR-1TK^−/−^ mice neither alter the proliferative activity of GFAP+ cells nor augment the generation of mature GFAP+ cells in the SVZ/RMS/OB. At the same time, proliferative DCX−/GFAP− cells have been significantly reduced. The elevated levels of endogenous VEGF-A in these mice seem to selectively activate NPC proliferation in the adult SVZ. In line with this, VEGFR-1TK^−/−^ mice and mice that received ectopic VEGF-A intracerebroventriculary displayed an elevated phosphorylation of VEGFR-2 in migrating DCX+ NPCs, indicating that VEGF-A directly augments NPCs migration and proliferation via VEGFR-2 [16]. Infusion of a specific VEGFR-2 inhibitor into the adult brain could elucidate the underlying signaling cascade in detail in vivo.

The increase of newly formed NPCs in the SVZ of VEGFR-1TK^−/−^ mice lead to more mature neurons in the OB, especially in the outer cell layers, the periglomerular layer, and the plexiform layer [16]. Conversely, the inhibition of VEGF-A in adult mice did not alter the number of neurons in the OB, but affected dendritogenesis of newly formed neurons in the periglomerular layer [17]. This implies that VEGF-A is not essential for, but stimulates, adult olfactory neurogenesis in the adult OB [17].

In contrast to the observed specific stimulation of neuronal development after the intracerebroventricular delivery of VEGF-A [15, 16, 20], infusion of VEGF-A into the adult rat striatum strongly induced astroglial proliferation. Here, VEGFR-1, but not -2, was expressed in activated astrocytes [151].

VEGF-B that only binds VEGFR-1 and NRP-1 has also been identified as a neurotrophic factor that triggers NS/PCs proliferation in the SVZ and the hippocampus [21]. In compliance with this, VEGF-B^−/−^ mice display a reduced neurogenic activity in the adult brain. Intracerebral infusion of VEGF-B compensated for this effect and increased neurogenesis up to physiological level [21]. The absence of VEGFR-1 in NS/PCs in the rodent adult brain argues against a direct neurotrophic effect of VEGF-B in vivo. As VEGFR-1 is expressed on GFAP+ cells in regions of olfactory neurogenesis, these cells might transmit a mitogenic factor for NS/PCs in response to VEGF-B [16]. Furthermore, in vivo studies are needed to unravel the individual specific functions of VEGFR-1 and -2 in neural cell types.

##### VEGFR-1 and -2 regulate hippocampal neurogenesis and function

In the hippocampus, VEGF-A is coupled not only with neurogenesis but also with learning and memory. VEGF-A expression is upregulated during intensive memory tasks and in an enriched environment [155]. In turn, overexpression of VEGF-A in hippocampal neurons triggers neurogenesis and angiogenesis and leads to an improvement of cognitive capabilities. In line with this, the molecular inhibition of VEGF-A expression abolishes the environmental-induced boost of neurogenesis [155]. The positive effect of VEGF-A on hippocampal neurogenesis is likely mediated by VEGFR-2, as neural expression of a dominant-negative VEGFR-2 in the hippocampus reduces proliferative activity and learning ability. The hypothesis of a direct effect via neural-expressed VEGFR-2 is further supported by the fact that co-expression of a mutant form of VEGFR-2 antagonized VEGF-enhanced neurogenesis and learning without an affect on endothelial cell proliferation [155].

A recent study from Licht et al. [18] reported a regulatory function of VEGF-A in neuronal plasticity in the adult hippocampus. Inducible neuronal VEGF-A overexpression in a transgenic mouse model resulted in increased hippocampal angiogenesis, enhanced neurogenesis, and improved hippocampal-dependent contextual memory. In accordance with this, the loss of VEGF-A impaired memory without detectable effects on vascularization or neurogenesis. Furthermore, VEGF-A modulated long-term potentiation (LTP) in dentate gyrus granule cells [18]. It has yet not been clarified which receptor mediates this effect. In these mice, VEGFR-1 and -2 have not been detected on neuronal cells in wild-type or in reporter-mice, but Nrp-1 and -2 have been [18]. However, Nrps seem not to be able to transduce VEGF-A signals independently, due to their short intracellular domain lacking catalytic activity [10]. Unfortunately, the expression pattern of VEGFRs has not been examined in the VEGF overexpressing mouse model in this study.

In contrast to VEGF-A, the overexpression of PlGF, which selectively binds to VEGFR-1, but not -2, blocks learning capability and reduces neurogenesis [155]. This suggests that VEGFR-1 is a negative regulator of hippocampal neurogenesis similar to its role in RMS migration or endothelial proliferation.

VEGF-B, which also binds only to VEGFR-1 and Nrp-1, but not VEGFR-2, promotes proliferation of NPCs in the hippocampus [21] and points to a positive regulation via VEGFR-1.

Further studies are necessary in order to unravel the exact mechanism of hippocampal neurogenesis and learning and memory and the involvement of VEGFRs in a regulatory function.

#### Neuronal specification of specific subtypes: dopaminergic neurons

Besides its general role in the main regions of physiological adult neurogenesis, VEGF seems also to have a role in the development and maintenance of specific neuronal subtypes, specifically dopaminergic neurons. This hypothesis is supported by an earlier study from Silverman et al. [156], which demonstrates that VEGF-A stimulates the development of dopaminergic neurons in vitro in mesencephalic explants from prenatal rats. Likewise, the administration of VEGF-A to cultures of forming embryoid bodies from human embryonic stem cells notably fosters the production of neuroectodermal cells, which mostly differentiate into neurons that seem to retain the dopaminergic phenotype [157]. In cultures of fetal ventral mesencephalic cells, VEGF-A improved dopaminergic cell survival and increased neurite length [158]. In vivo data provide additional support for this idea. Neuron-specific inactivation of HIF-1α in mice leads to a reduction of dopaminergic neurons of the substantia nigra in the mutant compared to wild-type mice. This result has been ascribed to lower levels of VEGF-A protein in the brain tissue of these mice. Interestingly, dopaminergic cells in the ventral tegmental area remained unaffected [159]. Until now, it has not been elucidated whether the decrease in dopaminergic neurons is an adult-onset effect or whether VEGF-A influences the development of the dopaminergic system in vivo. Furthermore, the signaling pathway that is underlying this effect has not been shown. However, expression levels of VEGFR-1 and -2 in mice were significantly higher in mesencephalic NPCs compared with frontal NPCs. In HIF-1α-deficient mice, VEGFR-2 expression was increased in NPCs compared with controls [159]. VEGFR-1 has additionally been detected on mesencephalic astrocytes. Silverman et al. [156] showed that VEGF binding sites were densest on blood vessels and astroglial cells. Based on these findings, the authors hypothesized that neurogenic effects of VEGF on dopaminergic neurons in the mesencephalic explants are secondary effects of other trophic factors [156]. Glial cells that directly respond to VEGF are likely to secrete these survival- and proliferation-stimulating factors. VEGFR-1 is expressed in astroglial cells of the brain [16, 108] and might be a mediator of these VEGF-induced neurotrophic effects.

In contradiction, Studer et al. [160] reported that administration of VEGF-A recombinant protein or VEGF neutralizing antibody did not affect the generation of dopaminergic neurons in embryonic (E12) rat mesencephalon cell cultures under lowered oxygen or 20 % oxygen condition, although VEGF-A has been upregulated in lower oxygen condition in these cultures. No GFAP+ cells were detected in these mesencephalic cell cultures [160]. This could support the theory of a glial cell-mediated indirect neurotrophic effect of VEGF-A.

However, adult VEGFR-1TK^−/−^ mice, which show an increased amount of VEGF-A in the brain, have higher numbers of dopaminergic olfactory neurons in the periglomerular layer than control animals [16]. Until now, it is not clear if the different interneuron subtypes arise from one multi-potent precursor cell in the SVZ and if the final cell fate of the NPCs is (1) already determined prior to their migration through the RMS or (2) becomes specified during RMS migration, or (3) even not until their integration within the OB [135, 161–164]. Yet, in the RMS of the VEGFR-1TK^−/−^ mice, increased phosphorylation of VEGFR-2 on migrating NPCs has been detected, so there a direct effect of VEGF-A on the migrating NPCs, while an indirect effect on later fate determination within the OB is conceivable [16].

Besides VEGF-A signals, VEGFR-2 might likewise transmit neuroprotective signals of VEGF-C in primary E13.5 dopaminergic neurons. RNA of Vegfr-1 and -2 as well as nrp1 and nrp2 were abundantly expressed in embryonic midbrain cultures, but levels of vegfr-3 RNA were very low. However, transcription of *Vegfr*-*1* and -*3* increased after VEGF-C treatment, suggesting an effect of VEGF-C mediated by VEGFR-2 and/or VEGFR-3 [165].

In pathophysiological in vivo models of Parkinson’s disease, neuroprotective effects of VEGF-A and -B have been published [165–167]. Furthermore, VEGF-A is upregulated in the substantia nigra but not in the striatum of Parkinson’s disease patients [168]. This, together with the known possible vascular side effects, demonstrates the importance of elucidating the exact mechanism of neurotrophic properties of VEGF with regard to the development of therapeutic interventions.

### Effects of VEGFRs on neural cell migration in the developing and adult NS

#### VEGFRs trigger the migration of CNS cells

The motility of NSCs and their ability to migrate to a region of insult or growth is an essential property for the development, maintenance, and repair of the nervous system, and emphasizes their importance as therapeutic targets for neurodegenerative diseases or nervous system injury. VEGFRs mediate indirect and direct effects on neural migration and chemoattraction in the adult CNS and during development.

Multiple studies have shown a direct effect of VEGFs via VEGFRs in neural cell culture models. For example, VEGFR-2, but not VEGFR-1, facilitates VEGF-A-induced migration and chemoattraction of FGF-2-stimulated subventricular NS/PCs in vitro [107]. In a neurosphere model derived from neonatal SVZ, blockade of VEGF and inhibition of VEGFR-1-signaling by neutralizing antibodies resulted in a modified morphology of GFAP + cells and interfered with sphere formation and migration. Inhibition of VEGFR-2 decreased the migration capacity of these GFAP+ cells to a lesser extent, and concomitantly led to a reduction of VEGF-A expression [169].

One of the first studies showing an indirect effect of VEGF on neuronal migration in vivo is from Loissant et al. [12]. The authors demonstrated an indirect effect of VEGF-A on neuronal migration via VEGFR-2+ endothelial cells in the higher vocal center of adult canaries. A testosterone-secreting implant induced an increase in angiogenic activity and expression of VEGF-A in this brain region. VEGFR-2-expressing endothelial cells in turn produced the neurotrophic factor BDNF in response to VEGF-A. This was preceded by the recruitment of new neuronal cells into the higher vocal center of the neostriatum. VEGFR-2 inhibitor in vivo and in vitro blocked this mechanism [12].

We recently showed for the first time a physiological role for VEGFR-1 signaling and endogenous VEGF-A on the migration of VEGFR-2-expressing NPCs in the adult RMS in vivo [16]. In adult mice, we demonstrated that VEGFR-1 signaling negatively regulates NPC migration. VEGFR-1 is expressed in GFAP+ cells of the glial tube through which NPCs migrate on their way to the OB. Genetic inhibition of VEGFR-1 signaling in mice increased VEGF-A protein levels in the brain tissue, which resulted in an increased phosphorylation of VEGFR-2 in migrating NPCs. This effect was mimicked by intracerebroventricular infusion of VEGF-A. The enhanced migration towards the OB leads to an increased volume of the structure. In accordance with these findings, NS/PCs of SVZ-derived explants of these mice exhibited an enhanced migratory speed [16]. These data strongly suggest that interplay of VEGFRs expressed in different cell types regulate RMS migration, whereby astroglial VEGFR-1 most likely indirectly controls VEGFR-2 activity in NPCs via alternation of VEGF-A concentrations.

The vasculature has been shown to serve as a migration substrate for NPCs on their way to the OB. The sheathing endothelial cells and astrocytes of the RMS secrete signaling molecules that are crucial for the control of NPC migration [170, 171]. Bozoyan et al. [170] showed that astrocytic VEGF-A directs blood vessel development of the RMS and thereby affects neuroblasts migration during early postnatal development in vivo. Here, VEGF-A downregulation by miRNA in astrocytes in postnatal mice (P3-4) altered the development of the blood vessel scaffold in the RMS and reduced neuroblasts migration (P15-17) to the olfactory bulb. Licht et al. [17] demonstrated that sequestering of VEGF leads to collapse of the embryonic RMS vasculature and eliminates premature RMS migration. Whereas SVZ-derived neuroblasts of adult VEGFR-1-signaling-deficient mice, which exhibit elevated VEGF-A protein levels in vivo, migrate faster in explant culture [16], the addition of VEGF-A to postnatal slice cultures (P12–17) or the downregulation of VEGF-A in astrocytes did not alter neuroblast migration. This points towards a vasculature-dependent effect of VEGF-A in postnatal development [170]. Taken together, these findings suggest that VEGF-A is essential for embryonic, and strongly beneficial for adult, RMS migration.

The VEGF-A enhances neuroblast migration most likely through a direct mechanism via binding to VEGFR-2 in NPCs in the adult brain [16], but not during postnatal development [170]. VEGF-expression levels vary in astrocytes of the SVZ and RMS during postnatal development [170]. Consequently, different mechanisms of VEGF-A actions in the distinct developmental stages are conceivable. Snapyan et al. [171] have demonstrated that endothelial BDNF directly stimulates NPC migration. Therefore, it is conceivable that astrocytic VEGF-A acts directly on migrating NPCs via VEGFR-2, but also indirectly by stimulating endothelial cells to produce BDNF, which in turn additionally fosters NPC migration. Here again, in vivo blockade of VEGFR-2 in NPCs could probably answer the question if VEGF-A directly or indirectly controls RMS neuroblast migration.

VEGF additionally functions as a chemoattractive guidance molecule for cerebellar granule cells. These cells migrate from the external granule cell layer toward the Purkinje cell layer. Cerebellar granule cells express the VEGFR-2 and are guided by a VEGF-A gradient dependent on matrix-binding isoforms. Data from cerebellar slice cultures and in vivo experiments strongly suggest that VEGFR-2 mediates VEGF-A-guided granule cell migration [19]. The transmission of this effect is based on an interaction of VEGFR-2 and NMDARs. NMDARs are also expressed by migrating granule cells and control their migration. VEGFR-2 modifies NMDAR function and enhances the conductance and [Ca^2+^] influx of NMDAR in postnatal granule cell neurons prior to synaptogenesis, at a time when excitatory glutamatergic synaptic inputs on GCs have not yet been established [172]. A recent study from Liu et al. [173] compared chemotactic responses of NS/PCs to VEGF-A and downstream signaling pathways, using a murine NSC line C17.2 derived from the external germinal layer of the postnatal cerebellum. In these cells, chemoattraction towards VEGF-A changes and is dependent on the differentiation status of the cells.

Besides their role in NPC migration VEGFRs are regulators of neuronal patterning in conjunction with Nrp-Receptors. VEGFR-1 has been shown to interact with Nrp-1 in a medulloblastoma-derived NPC line transmitting VEGF-A signals fostering migration, survival, and proliferation. VEGF165 competitively inhibited Sema3a-induced cell migration and apoptosis by binding to Nrp-1, thereby blocking the formation of a signaling complex of NRP1 and VEGFR1 [174].

Moreover, VEGF-A has been shown to guide axon pathfinding of RGCs at the optic chiasm during development. Here, VEGF-A is expressed at the midline of the optic chiasm and acts via NRP1 in contralaterally projecting RGCs [175].

Likewise, the effects on neurons and astrocytes VEGFRs mediate migration of oligodendrocyte precursor cells (OPCs). Recently, Hayakawa et al. [176] reported a migration-promoting effect of VEGF-A on rat OPCs in vitro that is mediated via VEGFR-2. Unlike VEGF-C, which acts mitogenically on OPCs through VEGFR-3, VEGF-A did not affect OPC proliferation or differentiation [22, 176]. Furthermore, conditioned medium of cerebral endothelial cells increased OPC proliferation and migration, but only migration was blocked when VEGFR-2 signaling was inhibited, suggesting that migration and proliferation are regulated through different pathways involving different VEGF family members and VEGFRs [176].

#### VEGFRs influence the migration of PNS cells

In the PNS, interplay of Sema3a and VEGF-A regulates the migration of cells, cell somata of motor neurons, and axonal growth and pathfinding.

VEGFR-1, but not VEGFR-2, is expressed in motor neurons of the facial nerve, leading to the assumption that VEGF-A induces the formation of a complex of VEGFR-1 and Nrp-1 that then transmits the VEGF-A signal [177]. Furthermore, VEGF-A and Nrps-1 co-operate on the control of migration of neural crest cells invading the branchial arch during development in chick embryos. VEGFR-2 is also expressed in these cells and therefore likely to mediate VEGF-A signals through interaction with Nrp-1. Similar to the regulation of migration in motor neurons, the ligand Sema3A acts as a chemorepellent in neural crest cells [178]. Thus, the expression of VEGFRs together with their co-receptors for complex formation is another level of control in neuronal development.

VEGFRs and Nrps have also been shown to play an important role in axon growth at sympathetic neurovascular junctions that are critically involved in the determination of blood pressure and blood flow. At the neurovascular junctions in neonatal and adult rats, VEGF-A is expressed in vascular smooth muscle cells and cells of the arteries, whereas VEGF receptors NRP-1, VEGFR-1, and VEGFR-2 are expressed in postganglionic sympathetic neurons. VEGF-A blocks Sema3A-induced growth cone collapse in vitro, probably via NRP-1, and induces growth cone spreading through VEGFR-1 signaling. In vivo, the neutralization of VEGF hindered the re-innervation of denervated femoral arteries, implying that VEGF stimulates axons re-growth and/or directed the growth of axons to the denervated vessel [179].

Similarly, PlGF has been reported to influence PNS cell migration. PlGF is expressed in neuronal cells in sciatic nerves and lumbar DRGs, but not in myelating Schwann cells or in endothelial cells [149]. In peripheral neurons, PlGF-2 has been shown to have anti-chemorepulsive properties and to stimulate growth cone formation [180]. Nrp-1 has been suggested to transduce PlGF-2 chemoattraction, but the signaling co-receptor has not yet been identified.

Further experiments are needed to clarify the role of VEGFRs in PNS cell migration and axonal growth and pathfinding.

### Roles of VEGFRs in retinal development, maintenance and function

The vertebrate retina is a part of the CNS that is derived from the optic cup, which emerges from the neural tube at the region of the ventral diencephalon before the onset of neurogenesis. Cells from the luminar side of the optic cup form a VZ and show high mitogenic activity during development. Retinal progenitor cells (RPCs) are located near the ventricular surface, whereas postmitotic cells migrate to their site of differentiation thus forming the laminar structure of the retina [181]. The majority of retinal cell types (cones, ganglion cells, horizontal cells) develop prior to birth, but differentiation of the rods occurs postnatally [182] (for reviews, see [183, 184]). Interestingly, the vertebrate peripheral retina is still avascular at birth, and in mice vascularization takes place within the first 2 weeks of life. Therefore, the effects of VEGF on neural cells can be studied in this part of the murine retina during this time frame excluding vascular interactions [185].

#### VEGFR-1 and -2 in retinal embryonic development

During development, transcripts of *Vegfr*-*1* and -*2* are expressed in RPCs. Expression of VEGFR-2 mRNA in RPCs occurs throughout neuronal differentiation, but VEGFR-2 mRNA and protein levels in the retina fluctuate during development. Simultaneously, VEGF-A is expressed adjacent to the VEGFR-2 + cells, pointing towards a direct effect of VEGF-A in retina development [181]. In retinal pigment epithelial cells (RPEs), VEGF expression has been detected in the optic vesicle at E9.5, whereas VEGFR-2 was detected the earliest at P6.5 and increasing gradually until full expression was reached at P8.5 [186]. In vitro, VEGF increases the number of photoreceptor cells and rhodopsin protein levels. The mitogenic response to VEGF increases during development from E15 to P1 [182]. Furthermore, neutralization of VEGF-A increases apoptosis and lowers microvilli density and size [186].

Postnatal VEGFR-1 and -2 mRNA and protein have also been detected in non-vascular cells of the retina [182]. Saint-Geniez et al. [187] described astrocytes in the retinal ganglion cell (RGC) layer, the cells of inner nuclear layer, and Müller glial cells as VEGF-A-expressing cells. Later, during postnatal development (P17–33), the expression was associated with retinal vessels. The expression of VEGFRs begins in the superficial layer and proceeds to the deeper layers, followed by vascularization of the tissue region. Inhibition of VEGFRs during early postnatal retinal development on the peripheral, at that time avascular, retina induced a loss of cells in the inner nuclear layer where Müller cells reside and in the ganglion layer, in which astrocytes are also located. The mitogenic effect of VEGF-A might be further mediated by MAPK as a downstream target of VEGFRs [185]. In addition, Nishiguchi et al. [188] reported that VEGFR-2 is expressed in proliferating cells of the postnatal murine retina and that VEGF-A triggers the proliferation of RPCs through VEGFR2/Flk1 in explant cultures.

In accordance with these results in rodents, VEGFR-2 has been detected in RPCs in the VZ of the early postnatal chick retina, which develops completely avascular. VEGF-A is expressed by postmitotic neurons. Gain- and loss-of-function experiments revealed a proliferative response to VEGF-A that is mediated via VEGFR-2 in vitro and in vivo. While inducing RPC proliferation, VEGF-A suppresses the differentiation of cells. Knock-down experiments showed that VEGFR-2 is the mediator of both effects. Although VEGF-A acts through the same receptor, the downstream signaling pathways in both cell types are divergent. VEGF-induced proliferation of RPCs was dependent on MEK–ERK activation, whereas the inhibitory effect on RGC differentiation was independent of this pathway. Interestingly, amacrine cell differentiation at the same time was not affected, suggesting cell-type-specific responsiveness to VEGF-A signals [189].

#### Neurotrophic effects of VEGFR-1 and -2 in the adult retina

In vivo, VEGF-A is expressed by RPEs as well as by retinal neurons and glial cells [190, 191]. VEGFR-2 and VEGFR-1 expression was detected in adult photoreceptor and Müller glia and vascular cells [187]. VEGFR-2 has also been detected in RGCs of the adult retina [192, 193]. Müller cells additionally express Nrp-1. The expression of three receptors for VEGF in Müller glia points to an autocrine effect of VEGFs in vivo that has been demonstrated in a Müller glia cell line [187].

Conflicting results exist concerning the effect of VEGFs on adult retinal cell survival. Two studies have shown that in vivo inhibition of VEGF signaling or sequestering of VEGFs, similar to the treatment of degenerative eye diseases due to vascular overgrowth, does not change photoreceptor or ganglionic cell viability or alter retinal function in adult and juvenile mice [190, 191]. In contrast, Saint-Geniez et al. [187] reported that systemic VEGF neutralization in adult mice via an adenovirus expressing sVEGFR-1 did not alter the retinal vascularization, but induced massive cell death in Müller cells, and amacrine and photoreceptor cells, which led to visual dysfunction. From the same laboratory, Ford et al. [186] reported degenerative changes of RPEs in the same mouse model after systemic VEGF-A neutralization. In photoreceptors, VEGF-A acted neuroprotectively against cell death induced by serum starvation. VEGFR-2 is constantly active in the adult retina, which points to a role in retinal homeostasis. In accordance with this, systemic blockade of VEGF for 8 weeks in mice caused loss of RGC [187].

Under pathophysiological conditions, VEGFR-2, but not VEGFR-1, signaling has been suggested to mediate retinal neuroprotection during ischemic injury [194]. This is supported by a study of Böcker-Meffert et al. [195], which demonstrates that VEGFR-2 mediates VEGF-A-stimulated neurite regrowth of axotomized RGCs in retinal explant cultures of postnatal rats (P11). In contrast, Li et al. [144] demonstrated expression and function of VEGFR-1 in retinal neuroprotection in a pathophysiological model. Blockade of VEGFR-1 eliminates the inhibitory effect of VEGF-B on the expression of anti-apoptotic genes in RGCs, strongly suggesting that VEGFR-1 mediates the neuroprotective properties of VEGF-B in the retina. In contradiction to VEGF-A, VEGF-B lacks a general angiogenic activity (except in the heart), which makes it an ideal candidate for neuroprotective therapies without affecting the vascular system [89].

In summary, the different VEGF family members and VEGFRs exert distinct functions in the retina. Whereas VEGFR-2 appears to be the main receptor facilitating mitogenic effects of VEGF-A during retinal development, both VEGFRs mediate VEGF-induced neuroprotection in the adult retina.

## Conclusions and outlook

The VEGF/VEGFR signaling system, initially discovered in the vascular system and thought to act specifically in the vascular system, clearly plays an important role in neural development, neural homeostasis, and recovery after neuronal injury. The findings that VEGF-A has a direct effect on neural progenitors as well as on differentiated neuronal cells, and may promote neurogenesis, neural migration, neuronal survival, and differentiation, has paved the way for novel therapeutic strategies. Currently, VEGF-A treatment is in clinical trial for patients with neurodegenerative disease (AML). These and future clinical studies will finally show the potency of VEGF family molecules in treatments for neurological disorders.

## References

[1] Nern C, Momma S (2006). The realized niche of adult neural stem cells. Stem Cell Rev.

[2] Lathia JD (2007). The microenvironment of the embryonic neural stem cell: lessons from adult niches?. Dev Dyn.

[3] Tavazoie M (2008). A specialized vascular niche for adult neural stem cells. Cell Stem Cell.

[4] Schlessinger J, Ullrich A (1992). Growth factor signaling by receptor tyrosine kinases. Neuron.

[5] Hausott B (2009). Signaling by neuronal tyrosine kinase receptors: relevance for development and regeneration. Anat Rec (Hoboken).

[6] Obermeier A (1995). Definition of signals for neuronal differentiation. Ann NY Acad Sci.

[7] Shibuya M (1990). Nucleotide sequence and expression of a novel human receptor-type tyrosine kinase gene (flt) closely related to the fms family. Oncogene.

[8] Vries C (1992). The fms-like tyrosine kinase, a receptor for vascular endothelial growth factor. Science.

[9] Terman BI (1991). Identification of a new endothelial cell growth factor receptor tyrosine kinase. Oncogene.

[10] Takahashi H, Shibuya M (2005). The vascular endothelial growth factor (VEGF)/VEGF receptor system and its role under physiological and pathological conditions. Clin Sci.

[11] Matsumoto T (2001) VEGF Receptor Signal Transduction. Sci STKE p 112:re–21 10.1126/stke.2001.112.re21 11741095

[12] Louissaint A (2002). Coordinated interaction of neurogenesis and angiogenesis in the adult songbird brain. Neuron.

[13] Sondell M (1999). Vascular endothelial growth factor has neurotrophic activity and stimulates axonal outgrowth, enhancing cell survival and Schwann cell proliferation in the peripheral nervous system. J Neurosci.

[14] Palmer TD (2000). Vascular niche for adult hippocampal neurogenesis. J Comp Neurol.

[15] Schänzer A (2004). Direct stimulation of adult neural stem cells in vitro and neurogenesis in vivo by vascular endothelial growth factor. Brain Pathol.

[16] Wittko IM (2009). VEGFR-1 regulates adult olfactory bulb neurogenesis and migration of neural progenitors in the rostral migratory stream in vivo. J Neurosci.

[17] Licht T (2009). VEGF is required for dendritogenesis of newly born olfactory bulb interneurons. Development.

[18] Licht T (2011). Reversible modulations of neuronal plasticity by VEGF. Proc Nat Acad Sci USA.

[19] Ruiz de Almodovar C (2010). Matrix-binding vascular endothelial growth factor (VEGF) isoforms guide granule cell migration in the cerebellum via VEGF receptor Flk1. J Neurosci.

[20] Jin K (2002). Vascular endothelial growth factor (VEGF) stimulates neurogenesis in vitro and in vivo. Proc Natl Acad Sci USA.

[21] Sun Y (2006). Vascular endothelial growth factor-B (VEGFB) stimulates neurogenesis: Evidence from knockout mice and growth factor administration. Dev Biol.

[22] Le Bras B (2006). VEGF-C is a trophic factor for neural progenitors in the vertebrate embryonic brain. Nat Neurosci.

[23] Falk T (2011). Vascular endothelial growth factor-B is neuroprotective in an in vivo rat model of Parkinson’s disease. Neurosci Lett.

[24] Calvo C-F (2011). Vascular endothelial growth factor receptor 3 directly regulates murine neurogenesis. Genes Dev.

[25] Ferrara N (2003). The biology of VEGF and its receptors. Nat Med.

[26] Carmeliet P (2003). Blood vessels and nerves: common signals, pathways and diseases. Nat Rev Genet.

[27] Raab S, Plate KH (2007). Different networks, common growth factors: shared growth factors and receptors of the vascular and the nervous system. Acta Neuropathol.

[28] Ruiz de Almodovar C (2009). Role and therapeutic potential of VEGF in the nervous system. Physiol Rev.

[29] Zhang ZG (2000). VEGF enhances angiogenesis and promotes blood-brain barrier leakage in the ischemic brain. J Clin Invest.

[30] van Bruggen N (1999). VEGF antagonism reduces edema formation and tissue damage after ischemia/reperfusion injury in the mouse brain. J Clin Invest.

[31] Palmer TD (2002). Adult neurogenesis and the vascular Nietzsche. Neuron.

[32] Ogunshola OO (2000). Neuronal VEGF expression correlates with angiogenesis in postnatal developing rat brain. Brain Res Dev Brain Res.

[33] Shen Q (2008). Adult SVZ stem cells lie in a vascular niche: a quantitative analysis of niche cell-cell interactions. Cell Stem Cell.

[34] Lee J (1996). Vascular endothelial growth factor-related protein: a ligand and specific activator of the tyrosine kinase receptor Flt4. Proc Nat Acad Sci USA.

[35] Joukov V (1996). A novel vascular endothelial growth factor, VEGF-C, is a ligand for the Flt4 (VEGFR-3) and KDR (VEGFR-2) receptor tyrosine kinases. The EMBO J.

[36] Koch S, Claesson-Welsh L (2012). Signal transduction by vascular endothelial growth factor receptors. Cold Spring Harb Perspect Med.

[37] Olsson A-K (2006). VEGF receptor signalling—in control of vascular function. Nat Rev Mol Cell Biol.

[38] Kendall RL (1996). Identification of a natural soluble form of the vascular endothelial growth factor receptor, FLT-1, and its heterodimerization with KDR. Biochem Biophys Res Commun.

[39] Shiose S (2000). Gene transfer of a soluble receptor of VEGF inhibits the growth of experimental eyelid malignant melanoma. Invest Ophthalmol Vis Sci.

[40] Chen H (2000). Inhibition of vascular endothelial growth factor activity by transfection with the soluble FLT-1 gene. J Cardiovasc Pharmacol.

[41] Waltenberger J (1994). Different signal transduction properties of KDR and Flt1, two receptors for vascular endothelial growth factor. J Biol Chem.

[42] Shibuya M (1999). Structure and function of vascular endothelial growth factor receptor-1 and -2. Curr Top Microbiol Immunol.

[43] Tchaikovski V (2008). The molecular basis of VEGFR-1 signal transduction pathways in primary human monocytes. Arterioscler Thromb Vasc Biol.

[44] Kanno S (2000). Roles of two VEGF receptors, Flt-1 and KDR, in the signal transduction of VEGF effects in human vascular endothelial cells. Oncogene.

[45] Fong GH (1996). Regulation of flt-1 expression during mouse embryogenesis suggests a role in the establishment of vascular endothelium. Dev Dyn.

[46] Fong GH (1995). Role of the Flt-1 receptor tyrosine kinase in regulating the assembly of vascular endothelium. Nature.

[47] Fong GH (1999). Increased hemangioblast commitment, not vascular disorganization, is the primary defect in flt-1 knock-out mice. Development.

[48] Hiratsuka S (2005). Membrane fixation of vascular endothelial growth factor receptor 1 ligand-binding domain is important for vasculogenesis and angiogenesis in mice. Mol Cell Biol.

[49] Hiratsuka S (1998). Flt-1 lacking the tyrosine kinase domain is sufficient for normal development and angiogenesis in mice. Proc Nat Acad Sci USA.

[50] Hiratsuka S (2001). Involvement of Flt-1 tyrosine kinase (vascular endothelial growth factor receptor-1) in pathological angiogenesis. Cancer Res.

[51] Albuquerque RJC (2009). Alternatively spliced vascular endothelial growth factor receptor-2 is an essential endogenous inhibitor of lymphatic vessel growth. Nat Med.

[52] Shalaby F (1997). A requirement for Flk1 in primitive and definitive hematopoiesis and vasculogenesis. Cell.

[53] Shalaby F (1995). Failure of blood-island formation and vasculogenesis in Flk-1-deficient mice. Nature.

[54] Kremer C (1997). Up-regulation of flk-1/vascular endothelial growth factor receptor 2 by its ligand in a cerebral slice culture system. Cancer Res.

[55] Schwarz Q, Ruhrberg C (2010). Neuropilin, you gotta let me know: should I stay or should I go?. Cell Adh Migr.

[56] Fujisawa H (1997). Roles of a neuronal cell-surface molecule, neuropilin, in nerve fiber fasciculation and guidance. Cell Tissue Res.

[57] Soker S (2002). VEGF165 mediates formation of complexes containing VEGFR-2 and neuropilin-1 that enhance VEGF165-receptor binding. J Cell Biochem.

[58] Favier B (2006). Neuropilin-2 interacts with VEGFR-2 and VEGFR-3 and promotes human endothelial cell survival and migration. Blood.

[59] Fuh G (2000). The interaction of neuropilin-1 with vascular endothelial growth factor and its receptor flt-1. J Biol Chem.

[60] Kitsukawa T et al (1995) Overexpression of a membrane protein, neuropilin, in chimeric mice causes anomalies in the cardiovascular system, nervous system and limbs. Development (Cambridge, England) 121:4309–4318 10.1242/dev.121.12.4309 8575331

[61] Kawasaki T (1999). A requirement for neuropilin-1 in embryonic vessel formation. Development.

[62] Yuan L (2002). Abnormal lymphatic vessel development in neuropilin 2 mutant mice. Development.

[63] Senger DR (1983). Tumor cells secrete a vascular permeability factor that promotes accumulation of ascites fluid. Science.

[64] Robinson CJ, Stringer SE (2001). The splice variants of vascular endothelial growth factor (VEGF) and their receptors. J Cell Sci.

[65] Ruhrberg C (2002). Spatially restricted patterning cues provided by heparin-binding VEGF-A control blood vessel branching morphogenesis. Genes Dev.

[66] Carmeliet P (1996). Abnormal blood vessel development and lethality in embryos lacking a single VEGF allele. Nature.

[67] Ferrara N (1996). Heterozygous embryonic lethality induced by targeted inactivation of the VEGF gene. Nature.

[68] Gerber HP (1999). VEGF is required for growth and survival in neonatal mice. Development.

[69] Gerhardt H (2003). VEGF guides angiogenic sprouting utilizing endothelial tip cell filopodia. J Cell Biol.

[70] Stalmans I (2002). Arteriolar and venular patterning in retinas of mice selectively expressing VEGF isoforms. J Clin Invest.

[71] Raab S (2004). Impaired brain angiogenesis and neuronal apoptosis induced by conditional homozygous inactivation of vascular endothelial growth factor. Thromb Haemost.

[72] Lee S (2007). Autocrine VEGF signaling is required for vascular homeostasis. Cell.

[73] Forsythe JA (1996). Activation of vascular endothelial growth factor gene transcription by hypoxia-inducible factor 1. Mol Cell Biol.

[74] Liu Y (1995). Hypoxia regulates vascular endothelial growth factor gene expression in endothelial cells. Identification of a 5’ enhancer. Circ Res.

[75] Levy AP (1995). Transcriptional regulation of the rat vascular endothelial growth factor gene by hypoxia. J Biol Chem.

[76] Plate KH (1993). Up-regulation of vascular endothelial growth factor and its cognate receptors in a rat glioma model of tumor angiogenesis. Cancer Res.

[77] Plate KH (1992). Vascular endothelial growth factor is a potential tumour angiogenesis factor in human gliomas in vivo. Nature.

[78] Krum JM, Rosenstein JM (1999). Transient coexpression of nestin, GFAP, and vascular endothelial growth factor in mature reactive astroglia following neural grafting or brain wounds. Exp Neurol.

[79] Plate KH (1999). Cell type specific upregulation of vascular endothelial growth factor in an MCA-occlusion model of cerebral infarct. J Neuropathol Exp Neurol.

[80] Oosthuyse B (2001). Deletion of the hypoxia-response element in the vascular endothelial growth factor promoter causes motor neuron degeneration. Nat Genet.

[81] Olofsson B (1996). Genomic organization of the mouse and human genes for vascular endothelial growth factor B (VEGF-B) and characterization of a second splice isoform. J Biol Chem.

[82] Olofsson B (1996). Vascular endothelial growth factor B, a novel growth factor for endothelial cells. Proc Nat Acad Sci USA.

[83] Silvestre J-S (2003). Vascular endothelial growth factor-B promotes in vivo angiogenesis. Circ Res.

[84] Aase K (2001). Vascular endothelial growth factor-B-deficient mice display an atrial conduction defect. Circulation.

[85] Bellomo D (2000). Mice lacking the vascular endothelial growth factor-B gene (Vegfb) have smaller hearts, dysfunctional coronary vasculature, and impaired recovery from cardiac ischemia. Circ Res.

[86] Wanstall JC (2002). Vascular endothelial growth factor-B-deficient mice show impaired development of hypoxic pulmonary hypertension. Cardiovasc Res.

[87] Enholm B (1997). Comparison of VEGF, VEGF-B, VEGF-C and Ang-1 mRNA regulation by serum, growth factors, oncoproteins and hypoxia. Oncogene.

[88] Zhang F (2009). VEGF-B is dispensable for blood vessel growth but critical for their survival, and VEGF-B targeting inhibits pathological angiogenesis. Proc Nat Acad Sci USA.

[89] Li X (2009). VEGF-B: a survival, or an angiogenic factor?. Cell Adh Migr.

[90] Falk T (2010). The Yin and Yang of VEGF and PEDF: Multifaceted Neurotrophic Factors and Their Potential in the Treatment of Parkinson’s Disease. Int J Mol Sci.

[91] Maglione D (1991). Isolation of a human placenta cDNA coding for a protein related to the vascular permeability factor. Proc Nat Acad Sci USA.

[92] DiPalma T (1996). The placenta growth factor gene of the mouse. Mammal Genome.

[93] Carmeliet P (2001). Synergism between vascular endothelial growth factor and placental growth factor contributes to angiogenesis and plasma extravasation in pathological conditions. Nat Med.

[94] Odorisio T (2002). Mice overexpressing placenta growth factor exhibit increased vascularization and vessel permeability. J Cell Sci.

[95] Freitas-Andrade M (2012). PlGF knockout delays brain vessel growth and maturation upon systemic hypoxic challenge. J Cereb Blood Flow Metab.

[96] Temple S (2001). The development of neural stem cells. Nature.

[97] McKay RD (2004). Stem cell biology and neurodegenerative disease. Philos Trans R Soc B.

[98] Weiss S (1996). Is there a neural stem cell in the mammalian forebrain?. Trends Neurosci.

[99] McKay R (1997). Stem cells in the central nervous system. Science.

[100] Breier G (1992). Expression of vascular endothelial growth factor during embryonic angiogenesis and endothelial cell differentiation. Development.

[101] Breier G (1995). Coordinate expression of vascular endothelial growth factor receptor-1 (flt-1) and its ligand suggests a paracrine regulation of murine vascular development. Dev Dyn.

[102] Haigh JJ (2003). Cortical and retinal defects caused by dosage-dependent reductions in VEGF-A paracrine signaling. Dev Biol.

[103] Acker T (2001). Cell type specific expression of vascular endothelial growth factor and angiopoietin-1 and -2 suggests an important role of astrocytes in cerebellar vascularization. Mechan Dev.

[104] Lagercrantz J (1998). A comparative study of the expression patterns for vegf, vegf-b/vrf and vegf-c in the developing and adult mouse. Biochim Biophys Acta.

[105] Ogunshola OO (2002). Paracrine and autocrine functions of neuronal vascular endothelial growth factor (VEGF) in the central nervous system. J Biol Chem.

[106] Bellon A (2010). VEGFR2 (KDR/Flk1) signaling mediates axon growth in response to semaphorin 3E in the developing brain. Neuron.

[107] Zhang H (2003). VEGF is a chemoattractant for FGF-2-stimulated neural progenitors. J Cell Biol.

[108] Mani N (2005). Astrocyte growth effects of vascular endothelial growth factor (VEGF) application to perinatal neocortical explants: receptor mediation and signal transduction pathways. Exp Neurol.

[109] Yang S-Z (2003). Distribution of Flk-1 and Flt-1 receptors in neonatal and adult rat brains. Anat Rec.

[110] Jin K (2006). Vascular endothelial growth factor stimulates neurite outgrowth from cerebral cortical neurons via Rho kinase signaling. J Neurobiol.

[111] Zhu Y (2003). Vascular endothelial growth factor promotes proliferation of cortical neuron precursors by regulating E2F expression. FASEB J.

[112] Zachary IC (2011). How neuropilin-1 regulates receptor tyrosine kinase signalling: the knowns and known unknowns. Biochem Soc Trans.

[113] Du H (2010). Vascular endothelial growth factor signaling implicated in neuroprotective effects of placental growth factor in an in vitro ischemic model. Brain Res.

[114] Rosenstein JM (2003). Neurotrophic effects of vascular endothelial growth factor on organotypic cortical explants and primary cortical neurons. J Neurosci.

[115] Sondell M (2000). Vascular endothelial growth factor is a neurotrophic factor which stimulates axonal outgrowth through the flk-1 receptor. Eur J Neurosci.

[116] Learte AR (2007). Gliatrophic and gliatropic roles of PVF/PVR signaling during axon guidance. Glia.

[117] Altman J, Das GD (1965). Autoradiographic and histological evidence of postnatal hippocampal neurogenesis in rats. J Comp Neurol.

[118] Cameron HA (1993). Differentiation of newly born neurons and glia in the dentate gyrus of the adult rat. Neuroscience.

[119] Lois C, Alvarez-Buylla A (1993). Proliferating subventricular zone cells in the adult mammalian forebrain can differentiate into neurons and glia. Proc Natl Acad Sci USA.

[120] Luskin MB (1993). Restricted proliferation and migration of postnatally generated neurons derived from the forebrain subventricular zone. Neuron.

[121] Doetsch F (1999). Subventricular zone astrocytes are neural stem cells in the adult mammalian brain. Cell.

[122] Doetsch F (1997). Cellular composition and three-dimensional organization of the subventricular germinal zone in the adult mammalian brain. J Neurosci.

[123] Lois C, Alvarez-Buylla A (1994). Long-distance neuronal migration in the adult mammalian brain. Science.

[124] Lois C (1996). Chain migration of neuronal precursors. Science.

[125] Nadarajah B, Parnavelas JG (2002). Modes of neuronal migration in the developing cerebral cortex. Nat Rev Neurosci.

[126] Menezes JR (1995). The division of neuronal progenitor cells during migration in the neonatal mammalian forebrain. Mol Cell Neurosci.

[127] Ma DK (2005). Glial influences on neural stem cell development: cellular niches for adult neurogenesis. Curr Opin Neurobiol.

[128] Lemke G (2001). Glial control of neuronal development. Annu Rev Neurosci.

[129] Sobeih MM, Corfas G (2002). Extracellular factors that regulate neuronal migration in the central nervous system. Int J Dev Neurosci.

[130] Bovetti S (2007). Blood vessels form a scaffold for neuroblast migration in the adult olfactory bulb. J Neurosci.

[131] Bovetti S (2007). Subventricular zone-derived neuroblast migration to the olfactory bulb is modulated by matrix remodelling. Eur J Neurosci.

[132] Carleton A (2003). Becoming a new neuron in the adult olfactory bulb. Nat Neurosci.

[133] Carlén M (2002). Functional integration of adult-born neurons. Curr Biol.

[134] Winner B (2002). Long-term survival and cell death of newly generated neurons in the adult rat olfactory bulb. Eur J Neurosci.

[135] Bovetti S (2007). Spatio-temporal specification of olfactory bulb interneurons. J Mol Hist.

[136] Androutsellis-Theotokis A (2009). Targeting neural precursors in the adult brain rescues injured dopamine neurons. Proc Nat Acad Sci USA.

[137] Harms KM (2010). Murine neural stem/progenitor sells protect neurons against ischemia by HIF-1αregulated VEGF signaling. PLoS ONE.

[138] Marti HH, Risau W (1998). Systemic hypoxia changes the organ-specific distribution of vascular endothelial growth factor and its receptors. Proc Natl Acad Sci USA.

[139] Balenci L (2007). IQGAP1 regulates adult neural progenitors in vivo and vascular Endothelial growth factor-triggered neural progenitor migration in vitro. J Neurosci.

[140] Ng YS (2001). Differential expression of VEGF isoforms in mouse during development and in the adult. Dev Dyn.

[141] Meng H (2006). Biphasic effects of exogenous VEGF on VEGF expression of adult neural progenitors. Neurosci Lett.

[142] Maurer MH (2003). Expression of vascular endothelial growth factor and its receptors in rat neural stem cells. Neurosci Lett.

[143] Nag S (2002). Differential expression of vascular endothelial growth factor-A (VEGF-A) and VEGF-B after brain injury. J Neuropathol Exp Neurol.

[144] Li Y (2008). VEGF-B inhibits apoptosis via VEGFR-1-mediated suppression of the expression of BH3-only protein genes in mice and rats. J Clin Invest.

[145] Sun Y (2004). Increased severity of cerebral ischemic injury in vascular endothelial growth factor-B-deficient mice. J Cereb Blood Flow Metab.

[146] Falk T (2009). Vascular endothelial growth factor B (VEGF-B) is upregulated and exogenous VEGF-B is neuroprotective in a culture model of Parkinson’s disease. Mol Neurodegener.

[147] Poesen K (2008). Novel role for vascular endothelial growth factor (VEGF) receptor-1 and its ligand VEGF-B in motor neuron degeneration. J Neurosci.

[148] Beck H (2002). Cell type-specific expression of neuropilins in an MCA-occlusion model in mice suggests a potential role in post-ischemic brain remodeling. J Neuropathol Exp Neurol.

[149] Chaballe L (2011). Involvement of placental growth factor in Wallerian degeneration. Glia.

[150] Thomas LB (1996). Young neurons from the adult subependymal zone proliferate and migrate along an astrocyte, extracellular matrix-rich pathway. Glia.

[151] Krum JM (2002). Angiogenic and astroglial responses to vascular endothelial growth factor administration in adult rat brain. Neuroscience.

[152] Choi J-S (2007). Upregulation of vascular endothelial growth factor receptors Flt-1 and Flk-1 in rat hippocampus after transient forebrain ischemia. J Neurotrauma.

[153] Choi J-S (2007). Upregulation of vascular endothelial growth factor receptors Flt-1 and Flk-1 following acute spinal cord contusion in rats. J Histochem Cytochem.

[154] Tonchev AB (2007). Expression of angiogenic and neurotrophic factors in the progenitor cell niche of adult monkey subventricular zone. Neuroscience.

[155] Cao L (2004). VEGF links hippocampal activity with neurogenesis, learning and memory. Nat Genet.

[156] Silverman WF (1999). Vascular, glial and neuronal effects of vascular endothelial growth factor in mesencephalic explant cultures. Neuroscience.

[157] Kim B-K (2006). Neurogenic effect of vascular endothelial growth factor during germ layer formation of human embryonic stem cells. FEBS Lett.

[158] Pitzer MR (2003). Angiogenic and neurotrophic effects of vascular endothelial growth factor (VEGF165): studies of grafted and cultured embryonic ventral mesencephalic cells. Exp Neurol.

[159] Milosevic J (2007). Lack of hypoxia-inducible factor-1 impairs midbrain neural precursor cells involving vascular endothelial growth factor signaling. J Neurosci.

[160] Studer L (2000). Enhanced proliferation, survival, and dopaminergic differentiation of CNS precursors in lowered oxygen. J Neurosci.

[161] Baker H (2001). Phenotypic differentiation during migration of dopaminergic progenitor cells to the olfactory bulb. J Neurosci.

[162] Hack MA (2005). Neuronal fate determinants of adult olfactory bulb neurogenesis. Nat Neurosci.

[163] Merkle FT (2007). Mosaic organization of neural stem cells in the adult brain. Science.

[164] De Marchis S (2004). GABAergic phenotypic differentiation of a subpopulation of subventricular derived migrating progenitors. Eur J Neurosci.

[165] Piltonen M (2011). Vascular endothelial growth factor C acts as a neurotrophic factor for dopamine neurons in vitro and in vivo. Neuroscience.

[166] Yasuhara T (2005). Neurorescue effects of VEGF on a rat model of Parkinson’s disease. Brain Res.

[167] Yasuhara T (2004). Neuroprotective effects of vascular endothelial growth factor (VEGF) upon dopaminergic neurons in a rat model of Parkinson’s disease. Eur J Neurosci.

[168] Wada K (2006). Expression levels of vascular endothelial growth factor and its receptors in Parkinson´s disease. NeuroRep.

[169] Mani N (2010). Vascular endothelial growth factor enhances migration of astroglial cells in subventricular zone neurosphere cultures. J Neurosci Res.

[170] Bozoyan L (2012). Astrocytes control the development of the migration-promoting vasculature scaffold in the postnatal brain via VEGF signaling. J Neurosci.

[171] Snapyan M (2009). Vasculature guides migrating neuronal precursors in the adult mammalian forebrain via brain-derived neurotrophic factor signaling. J Neurosci.

[172] Meissirel C (2011). VEGF modulates NMDA receptors activity in cerebellar granule cells through Src-family kinases before synapse formation. Proc Nat Acad Sci USA.

[173] Liu J (2011). Differentiation of neural stem cells influences their chemotactic responses to vascular endothelial growth factor. J Neurosci Res.

[174] Bagnard D (2001). Semaphorin 3A-vascular endothelial growth factor-165 balance mediates migration and apoptosis of neural progenitor cells by the recruitment of shared receptor. J Neurosci.

[175] Erskine L (2011). VEGF signaling through neuropilin 1 guides commissural axon crossing at the optic chiasm. Neuron.

[176] Hayakawa K (2011). Vascular endothelial growth factor regulates the migration of oligodendrocyte precursor cells. J Neurosci.

[177] Schwarz Q (2004). Vascular endothelial growth factor controls neuronal migration and cooperates with Sema3A to pattern distinct compartments of the facial nerve. Genes Dev.

[178] McLennan R (2010). Vascular endothelial growth factor (VEGF) regulates cranial neural crest migration in vivo. Dev Biol.

[179] Marko SB, Damon DH (2008). VEGF promotes vascular sympathetic innervation. Am J Physiol Heart Circ Physiol.

[180] Cheng L (2004). Anti-chemorepulsive effects of vascular endothelial growth factor and placental growth factor-2 in dorsal root ganglion neurons are mediated via neuropilin-1 and cyclooxygenase-derived prostanoid production. J Biol Chem.

[181] Yang X, Cepko CL (1996). Flk-1, a receptor for vascular endothelial growth factor (VEGF), is expressed by retinal progenitor cells. J Neurosci.

[182] Yourey PA (2000). Vascular endothelial cell growth factors promote the in vitro development of rat photoreceptor cells. J Neurosci.

[183] Dowling JE (1987). The retina—an approachable part of the brain.

[184] Reese BE (2011). Development of the retina and optic pathway. Vis Res.

[185] Robinson GS (2001). Nonvascular role for VEGF: VEGFR-1, 2 activity is critical for neural retinal development. FASEB J.

[186] Ford KM (2011). Expression and role of VEGF in the adult retinal pigment epithelium. Invest Ophthalmol Vis Sci.

[187] Saint-Geniez M (2008). Endogenous VEGF is required for visual function: evidence for a survival role on müller cells and photoreceptors. PLoS ONE.

[188] Nishiguchi KM (2007). The role of VEGF and VEGFR2/Flk1 in proliferation of retinal progenitor cells in murine retinal degeneration. Invest Ophthalmol Vis Sci.

[189] Hashimoto T (2006). VEGF activates divergent intracellular signaling components to regulate retinal progenitor cell proliferation and neuronal differentiation. Development.

[190] Ueno S (2008). Prolonged blockade of VEGF family members does not cause identifiable damage to retinal neurons or vessels. J Cell Physiol.

[191] Miki A (2010). Prolonged blockade of VEGF receptors does not damage retinal photoreceptors or ganglion cells. J Cell Physiol.

[192] Kilic Ü (2006). Human vascular endothelial growth factor protects axotomized retinal ganglion cells in vivo by activating ERK-1/2 and Akt pathways. J Neurosci.

[193] Lindqvist N (2010). Multiple receptor tyrosine kinases are expressed in adult rat retinal ganglion cells as revealed by single-cell degenerate primer polymerase chain reaction. Uppsala J Med Sci.

[194] Nishijima K (2007). Vascular endothelial growth factor-A is a survival factor for retinal neurons and a critical neuroprotectant during the adaptive response to ischemic injury. Am J Pathol.

[195] Böcker-Meffert S (2002). Erythropoietin and VEGF promote neural outgrowth from retinal explants in postnatal rats. Invest Ophthalmol Vis Sci.

